# Effects of ketogenic diet on health outcomes: an umbrella review of meta-analyses of randomized clinical trials

**DOI:** 10.1186/s12916-023-02874-y

**Published:** 2023-05-25

**Authors:** Chanthawat Patikorn, Pantakarn Saidoung, Tuan Pham, Pochamana Phisalprapa, Yeong Yeh Lee, Krista A. Varady, Sajesh K. Veettil, Nathorn Chaiyakunapruk

**Affiliations:** 1 Department of Pharmacotherapy, College of Pharmacy, University of Utah, 30 2000 E, Salt Lake City, Utah 84112 USA; 2 Department of Social and Administrative Pharmacy, Faculty of Pharmaceutical Sciences, Chulalongkorn University, Bangkok, Thailand; 3 Division of Gastroenterology, Hepatology & Nutrition, Department of Internal Medicine, University of Utah, Salt Lake City, Utah USA; 4 Division of Ambulatory Medicine, Department of Medicine, Faculty of Medicine Siriraj Hospital, Mahidol University, Bangkok, Thailand; 5 School of Medical Sciences, Universiti Sains Malaysia, Kota Bharu, Malaysia; 6 Department of Kinesiology and Nutrition, University of Illinois at Chicago, Chicago, Illinois USA; 7 IDEAS Center, Veterans Affairs Salt Lake City Healthcare System, Salt Lake City, Utah USA

**Keywords:** Ketegenic diet, Umbrella review, Systematic review, Meta-analysis, Weight loss, Seizure

## Abstract

**Background:**

Systematic reviews and meta-analyses of randomized clinical trials (RCTs) have reported the benefits of ketogenic diets (KD) in various participants such as patients with epilepsy and adults with overweight or obesity**.** Nevertheless, there has been little synthesis of the strength and quality of this evidence in aggregate.

**Methods:**

To grade the evidence from published meta-analyses of RCTs that assessed the association of KD, ketogenic low-carbohydrate high-fat diet (K-LCHF), and very low-calorie KD (VLCKD) with health outcomes, PubMed, EMBASE, Epistemonikos, and Cochrane database of systematic reviews were searched up to February 15, 2023. Meta-analyses of RCTs of KD were included. Meta-analyses were re-performed using a random-effects model. The quality of evidence per association provided in meta-analyses was rated by the GRADE (Grading of Recommendations, Assessment, Development, and Evaluations) criteria as high, moderate, low, and very low.

**Results:**

We included 17 meta-analyses comprising 68 RCTs (median [interquartile range, IQR] sample size of 42 [20–104] participants and follow-up period of 13 [8–36] weeks) and 115 unique associations. There were 51 statistically significant associations (44%) of which four associations were supported by high-quality evidence (reduced triglyceride (*n* = 2), seizure frequency (*n* = 1) and increased low-density lipoprotein cholesterol (LDL-C) (*n* = 1)) and four associations supported by moderate-quality evidence (decrease in body weight, respiratory exchange ratio (RER), hemoglobin A_1c_, and increased total cholesterol). The remaining associations were supported by very low (26 associations) to low (17 associations) quality evidence. In overweight or obese adults, VLCKD was significantly associated with improvement in anthropometric and cardiometabolic outcomes without worsening muscle mass, LDL-C, and total cholesterol. K-LCHF was associated with reduced body weight and body fat percentage, but also reduced muscle mass in healthy participants.

**Conclusions:**

This umbrella review found beneficial associations of KD supported by moderate to high-quality evidence on seizure and several cardiometabolic parameters. However, KD was associated with a clinically meaningful increase in LDL-C. Clinical trials with long-term follow-up are warranted to investigate whether the short-term effects of KD will translate to beneficial effects on clinical outcomes such as cardiovascular events and mortality.

**Supplementary Information:**

The online version contains supplementary material available at 10.1186/s12916-023-02874-y.

## Background

Ketogenic diets (KD) have received substantial attention from the public primarily due to their ability to produce rapid weight loss in the short run [1, 2]. The KD eating pattern severely restricts carbohydrate intake to less than 50 g/day while increasing protein and fat intake [3–6]. Carbohydrate deprivation leads to an increase in circulating ketone bodies by breaking down fatty acids and ketogenic amino acids. Ketones are an alternative energy source from carbohydrates that alter physiological adaptations. These adaptions have been shown to produce weight loss with beneficial health effects by improving glycemic and lipid profiles [7, 8]. KD has also been recommended as a nonpharmacological treatment for medication-refractory epilepsy in children and adults [8, 9]. Evidence suggests that KD has reduced seizure frequency in patients with medication-refractory epilepsy, and even allowing some patients to reach complete and sustained remission.^11^ However, the exact anticonvulsive mechanism of KD remains unclear [10, 11].

Several systematic reviews and meta-analyses of randomized clinical trials (RCTs) have reported on the use of KD in patients with obesity or type 2 diabetes mellitus (T2DM) to control weight and improve cardiometabolic parameters [1, 12–15], in patients with refractory epilepsy to reduce seizure frequency [16], and in athletes to control weight and improve performance [17]. To date, there has been little synthesis of the strength and quality of this evidence in aggregate. This umbrella review therefore aims to systematically identify relevant meta-analyses of RCTs of KD, summarize their findings, and assess the strength of evidence of the effects of KD on health outcomes.

## Methods

The protocol of this study was registered with PROSPERO (CRD42022334717). We reported following the 2020 Preferred Reporting Items for Systematic Reviews and Meta-analyses (PRISMA) (Additional file 1) [18]. Difference from the original review protocol is described with rationale in Additional file 2: Table S1.

### Search strategy and eligibility criteria

We searched PubMed, EMBASE, Epistemonikos, and the Cochrane database of systematic reviews (CDSR) from the database inception to February 15, 2023 (Additional file 2: Table S2). No language restriction was applied. Study selection was independently performed in EndNote by two reviewers (C.P. and PS). After removing duplicates, the identified articles' titles and abstracts were screened for relevance. Full-text articles of the potentially eligible articles were retrieved and selected against the eligibility criteria. Any discrepancies were resolved by discussion with the third reviewer (SKV).

We included studies that met the following eligibility criteria: systematic reviews and meta-analyses of RCTs investigating the effects of any type of KD on any health outcomes in participants with or without any medical conditions compared with any comparators. When more than 1 meta-analysis was available for the same research question, we selected the meta-analysis with the largest data set [19–21]. Articles without full-text and meta-analyses that provided insufficient or inadequate data for quantitative synthesis were excluded.

### Data extraction and quality assessment

Two reviewers (CP and PS) independently performed data extraction and quality assessment (Additional file 2: Method S1). Discrepancies were resolved with consensus by discussing with the third reviewer (SKV). We used AMSTAR- 2 -A Measurement Tool to Assess Systematic Reviews- to grade the quality of meta-analyses as high, moderate, low, or critically low by assessing the following elements, research question, a priori protocol, search, study selection, data extraction, quality assessment, data analysis, interpretation, heterogeneity, publication bias, source of funding, conflict of interest [22].

### Data synthesis

For each association, we extracted effect sizes (mean difference [MD], the standardized mean difference [SMD], and risk ratio [RR]) of individual studies included in each meta-analysis and performed the meta-analyses to calculate the pooled effect sizes and 95% CIs using a random-effects model under DerSimonian and Laird [23], or the Hartung-Knapp- Sidik-Jonkman approach for meta-analyses with less than five studies [24]. *p* < 0.05 was considered statistically significant in 2-sided tests. Heterogeneity was evaluated using the *I*^*2*^ statistic. The evidence for small-study effects was assessed by the Egger regression asymmetry test [25]. Statistical analyses were conducted using Stata version 16.0 (StataCorp). We presented effect sizes of statistically significant associations with the known or estimated minimally clinically important difference (MCID) thresholds for health outcomes [14, 26–30].

We assessed the quality of evidence per association by applying the GRADE criteria (Grading of Recommendations, Assessment, Development, and Evaluations) in five domains, including (1) risk of bias in the individual studies, (2) inconsistency, (3) indirectness, (4) imprecision, and (5) publication bias [31]. We graded the strength of evidence (high, moderate, low, and very low) using GRADEpro version 3.6.1 (McMaster University).

### Sensitivity analyses

Sensitivity analyses were performed by excluding small-size studies (< 25^th^ percentile) [32] and excluding primary studies having a high risk of bias rated by the Cochrane’s risk of bias 2 tool (RoB 2) for RCTs from the identified associations [19–21, 33].

## Results

**Fig. 1:**
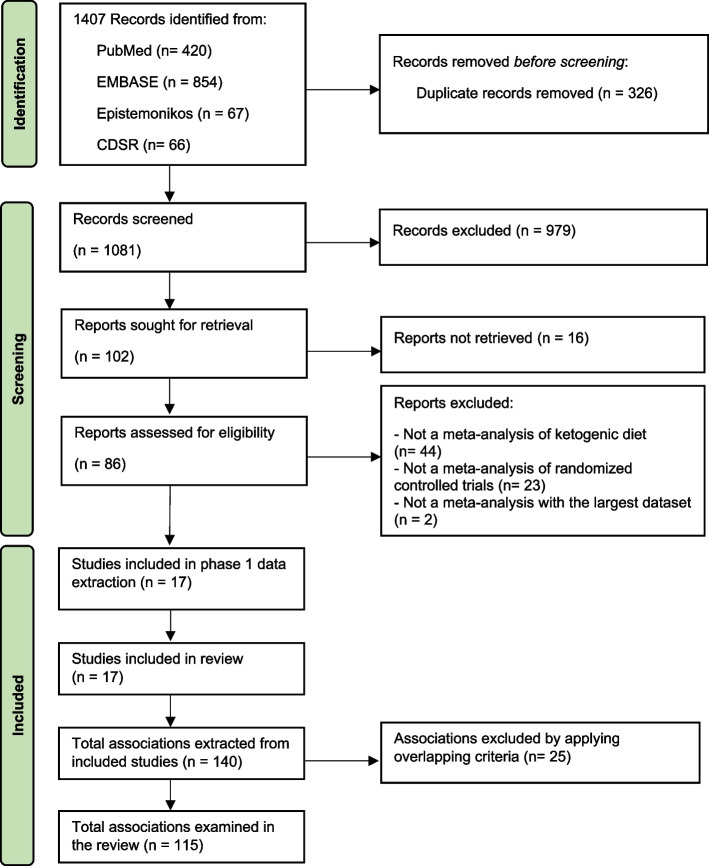
Study selection flow of meta-analyses. Abbreviation: CDSR, Cochrane database of systematic review

Seventeen meta-analyses were included (Fig. 1 and Additional file 2: Table S3) [1, 2, 15–17, 34–45]. These meta-analyses comprised 68 unique RCTs with a median (interquartile range, IQR) sample size per RCT of 42 (20–104) participants and a median (IQR) follow-up period of 13 (8–36) weeks. The quality of meta-analyses assessed using AMSTAR-2 found that none were rated as high confidence, 2 (12%) as moderate confidence, 2 (12%) as low confidence, and 13 (76.0%) as critically low confidence (Table 1 and Additional file 2: Table S4).

**Table 1:** Characteristics of meta-analyses of randomized clinical trials studying ketogenic diet

Source	Population	Type of KD	Comparator	Duration of diet	No. of included studies	Total participants	Outcomes	AMSTAR-2 rating
Alarim et al. 2020 [36]	T2DM adults with overweight or obesity	KD	LCD or RD	4–8 months	8	653	Body weight, BMI, FPG, HbA_1c_, LDL-C, HDL-C, TC, TG	Critically low
Amini et al. 2021 [37]	Individuals > 18 years old	KD or K-LCHF	LCD, LFD, HCD, or RD	2 weeks to 24 months	18	1835	Body weight, BMI, muscle mass, waist circumference, fat mass, body fat percentage, visceral adipose tissue,	Moderate
Ashtary-Larky et al. 2022 [38]	Individuals > 16 years old	KD or K-LCHF	RD or KD	3 weeks to 3 months	13	244	BMI, muscle mass, fat mass, body fat percentage	Critically low
Bueno et al. 2013 [1]	Adults with obesity	KD	LFD or RD	3–6 months	13	1415	Body weight, HDL-C, LDL-C, TG, FPG, HbA_1c_, fasting insulin, SBP, DBP, CRP	Moderate
Cao et al. 2021 [39]	Athletes	K-LCHF	HCD	4–6 weeks	10	139	VO_2_ max, maximal heart rate during exercise, respiratory exchange ratio	Critically low
Castellana et al. 2020 [15]	T2DM adults with overweight or obesity	VLCKD	LCD	1–2 months	13	801	Body weight	Low
Choi et al. 2020 [2]	Adults with overweight or obesity, some with T2DM	KD, K-LCHF, or VLCKD	LCD, LFD, HCD, or RD	120 min to 24 months	14	734	Body weight, BMI, waist circumference, HDL-C, LDL-C, TC, TG, FPG, HbA_1c_, fasting insulin, HOMA-IR, SBP, DBP, CRP, creatinine, C-peptide	Critically low
Lee et al. 2021 [40]	Adults with overweight or obesity	KD	RD	1–6 months	7	255	Body weight, BMI, fat mass, muscle mass, waist circumference, HDL-C, LDL-C, TC, TG, FPG, VO_2_ peak	Critically low
Lee et al. 2021 [17]	Athletes	KD	RD	2–24 weeks	8	158	Body fat percentage, muscle mass, HDL-C, TC, TG, FPG, fasting insulin, heart rate, VO_2_ max, respiratory exchange ratio,	Critically low
Lopez-Espinosa et al. 2021 [41]	Adults with obesity	KD, K-LCHF, or VLCKD	LCD, LFD, or HCD	24–96 weeks	10	943	BMI, HDL-C, LDL-C, TC, TG	Critically low
Muscogiuri et al. 2021 [42]	Adults with overweight or obesity	VLCKD	LCD	2–4 weeks	15	835	Body weight, BMI, fat mass, muscle mass, waist circumference, FPG, HbA_1C_, HOMA-IR, total cholesterol, triglyceride, HDL-C, LDL-C	Critically low
Rafiullah et al. 2022 [35]	T2DM adults	KD	RD	1–32 weeks	10	800	Body weight, LDL-C, TG, HbA_1c_	Critically low
Sainsbury et al. 2018 [34]	T2DM adults with overweight or obesity	KD	LCD or LFD	12–24 weeks	25	2784	HbA_1c_	Low
Smith et al. 2020[43]	Adults with obesity, some with dyslipidemia	KD	LDF or RD	12–96 weeks	25	3340	Muscle mass	Critically low
Sourbron et al. 2020 [16]	Children and adolescents (age 1–18 years) with refractory epilepsy	KD or MAD	RD	3–16 months	7	539	Seizure frequency reduction	Critically low
Vargas-Molina et al. 2022	Athletes or resistance trained adults	KD	RD	8–12 weeks	5	111	Body weight, muscle mass	Critically low
Yang et al. 2021 [44]	Adults with cancers	KD or K-LCHF	RD	6–24 weeks	6	325	Body weight, HDL-C, LDL-C, TC, TG, FPG, insulin, adverse effects	Critically low

*Abbreviations*: *AMSTAR-2* A Measurement Tool to Assess Systematic Reviews, *BMI* body mass index, *CRP* C-reactive protein, *DBP* diastolic blood pressure, *FPG* fasting plasma glucose, *HbA*_*1c*_ hemoglobin A_1c_, *HCD* high carbohydrate diet, *HDL-C* high-density lipoprotein cholesterol, *HOMA-IR* homeostatic model of insulin resistance, *K-LCHF* ketogenic low-carbohydrate high-fat diet, *KD* ketogenic diet, *LCD* low-calorie diet, *LDL-C* low-density lipoprotein cholesterol, *LFD* low-fat diet, *MAD* modified Atkins diet, *RD* regular diet, *SBP* systolic blood pressure, *T2DM* type 2 diabetic mellitus, *TC* total cholesterol, *TG* triglyceride, *VLCKD* very low-calorie ketogenic diet, *VO*_*2*_* max* maximum oxygen consumption, *VO*_*2*_* peak* peak oxygen consumption

Types of KD identified in this umbrella review were categorized as (1) KD, which limits carbohydrate intake to < 50 g/day or < 10% of the total energy intake (TEI) [35], (2) ketogenic low-carbohydrate, high-fat diet (K-LCHF), which limits carbohydrate intake to < 50 g/day or < 10% of TEI with high amount of fat intake (60–80% of TEI) [38, 46], (3) very low-calorie KD (VLCKD), which limits carbohydrate intake to < 30–50 g/day or 13–25% of TEI with TEI < 700–800 kcal/day, and (4) modified Atkins diet (MAD), which generally limits carbohydrate intake to < 10 g/day while encouraging high-fat foods [15, 47]. Meta-analyses of long-chain triglyceride KD, medium-chain triglyceride KD, and low glycemic index treatment were not identified.

### Description and summary of associations

We identified 115 unique associations of KD with health outcomes (Additional file 2: Table S5). The median (IQR) number of studies per association was 3 [4–6], and the median (IQR) sample size was 244 (127–430) participants. Outcomes were associated with KD types, including 40 (35%) KD, 18 (16%) K-LCHF, 13 (11%) VLCKD, 25 (22%) KD or K-LCHF, 5 (4%) KD or VLCKD, 1 (1%) KD or MAD, and 13 (11%) KD, K-LCHF, or VLCKD.

The associations involved 40 (35%) anthropometric measures (i.e., body weight, body mass index [BMI] [calculated as weight in kilograms divided by height in meters squared], waist circumference, muscle mass, fat mass, body fat percentage, and visceral adipose tissue), 37 (32%) lipid profile outcomes (i.e., triglyceride, total cholesterol, high-density lipoprotein cholesterol [HDL-C], and low-density lipoprotein cholesterol [LDL-C]), 22 (19%) glycemic profile outcomes (i.e., hemoglobin A_1c_ [HbA_1c_], fasting plasma glucose, fasting insulin, and homeostatic model assessment of insulin resistance [HOMA-IR]), 6 (5%) exercise performance (i.e., maximal heart rate, respiratory exchange ratio [RER], maximal oxygen consumption (VO_2_ max), 5 (4%) blood pressure outcomes (i.e., systolic blood pressure [SBP], diastolic blood pressure [DBP], and heart rate), 1 (1%) outcome associated with seizure frequency reduction ≥ 50% from baseline, and 3 other outcomes (i.e., serum creatinine, C-peptide, and C-reactive protein). In addition, there is 1 association (1%) of adverse events.

Participants in the identified associations included 68 (59%) associations in adults with overweight or obesity with or without T2DM or dyslipidemia, 15 (13%) athletes or resistance-trained adults, 12 (10%) adults with T2DM, 11 (10%) healthy participants ≥ 16 years old, 8 (7%) cancer patients, and 1 (1%) in children and adolescents with epilepsy.

Using GRADE, 115 associations were supported by very low strength of evidence (*n* = 66, 57%), with the remaining being low (*n* = 36, 31%), moderate (*n* = 9, 8%), and high quality of evidence (*n* = 4, 3%) (Additional file 2: Table S5). Almost half, or 44% (51 associations), were statistically significant based on a random-effects model, of which 51% (26 associations) were supported by a very low level of evidence, followed by low (17 associations [33%]), moderate (4 associations [8%]), and high (4 associations [8%]) levels of evidence. Overall beneficial outcomes associated with KD were BMI [37, 42], body weight [1, 2, 35–37, 41], waist circumference [37, 42], fat mass [37, 42], body fat percentage [38, 40], visceral adipose tissue [37], triglyceride [1, 2, 36, 42], HDL-C [1, 2, 42], HbA_1c _[2, 34, 35],  HOMA-IR [2, 42], DBP [1], seizure frequency reduction ≥ 50% from baseline [16], and respiratory exchange ratio [17, 39]. Adverse outcomes associated with KD were reduced muscle mass [37, 38], and increased LDL-C [2, 35], and total cholesterol [2, 17]. In terms of safety, one association showed no significant increase in adverse events (e.g., constipation, abdominal pain, and nausea) with KD [44].

Eight out of 13 associations supported by moderate to high-quality evidence were statistically significant (Table 2). There were 4 statistically significant associations supported by high-quality evidence, including the following: (1) KD or MAD for 3–16 months was associated with a higher proportion of children and adolescents with refractory epilepsy achieving seizure frequency reduction ≥ 50% from baseline compared with regular diet (RR, 5.11; 95% CI, 3.18 to 8.21) [16], (2) KD for 3 months was associated with reduced triglyceride in adults with T2DM compared with regular diet (MD, -18.36 mg/dL; 95% CI, -24.24 to -12.49, MCID threshold 7.96 mg/dL) [14, 35], (3) KD for 12 months was associated with reduced triglyceride in adults with T2DM compared with regular diet (MD, -24.10 mg/dL; 95% CI, -33.93 to -14.27, MCID threshold 7.96 mg/dL) [14, 35], and (4) KD for 12 months was associated with increased LDL-C in adults with T2DM compared with regular diet (MD, 6.35 mg/dL; 95% CI, 2.02 to 10.69, MCID threshold 3.87 mg/dL) [14, 35]. In addition, there were 4 statistically significant associations supported by moderate-quality evidence: (1) KD for 3 months was associated with reduced HbA_1c_ in adults with T2DM compared with regular diet (MD, -0.61%; 95% CI, -0.82 to -0.40, MCID threshold 0.5%) [14, 35], (2) VLCKD for 4–6 weeks was associated with reduced body weight in T2DM adults with overweight or obesity compared with a low-fat diet or regular diet (MD, -9.33 kg; 95% CI, -15.45 to -3.22, MCID threshold 4.40 kg) [14, 15], (3) K-LCHF for 4–6 weeks was associated with reduced respiratory exchange ratio in athletes compared with a high-carbohydrate diet (SMD, -2.66; 95% CI, -3.77 to -1.54) [39], and (4) K-LCHF for 11–24 weeks was associated with increased total cholesterol in athletes compared with regular diet (MD, 1.32 mg/dL; 95% CI, 0.64 to 1.99) [14, 17].

**Table 2:** Summary of significant associations of ketogenic diet with health outcomes supported by moderate to high quality of evidence

Source	Outcome	Population	Intervention	Duration of KD	Comparator	No. of studies (sample size)	Metric	Random effect size (95% CI)	*P* value	*I*^*2*^, %	GRADE rating	Clinical importance (MCID threshold)
Sourbron et al. 2020 [16]	Seizure frequency reduction ≥ 50% from baseline	Children or adolescents (age 1–18 years) with refractory epilepsy	KD or MAD	3–16 months	RD	5 (*n* = 374)	RR	5.11 (3.18 to 8.21)	< .001	0	High	N/A
Rafiullah et al. 2022 [35]	Triglyceride, mg/dL	Adults with T2DM	KD	3 months	RD	4 (*n* = 283)	MD	-18.36 (-24.24 to -12.49)	< .001	0	High	Yes (MCID 7.96 mg/dL)
Rafiullah et al. 2022 [35]	Triglyceride, mg/dL	Adults with T2DM	KD	12 months	RD	5 (*n* = 445)	MD	-24.10 (-33.93 to -14.27)	< .001	0	High	Yes (MCID 7.96 mg/dL)
Rafiullah et al. 2022 [35]	LDL-C, mg/dL	Adults with T2DM	KD	12 months	RD	4 (*n* = 389)	MD	6.35 (2.02 to 10.69)	.004	0	High	Yes (MCID 3.87 mg/dL)
Rafiullah et al. 2022 [35]	HbA_1c_, %	Adults with T2DM	KD	3 months	RD	6 (*n* = 388)	MD	-0.61 (-0.82 to -0.40)	< .001	44.0	Moderate	Yes (MCID 0.50%)
Castellana et al. 2020 [15]	Body weight, kg	T2DM adults with overweight or obesity	VLCKD	4–6 weeks	LFD or RD	2 (*n* = 142)	MD	-9.33 (-15.45 to -3.22)	< .001	0.1	Moderate	Yes (MCID 4.40 kg)
Cao et al. 2021 [39]	Respiratory exchange rate	Athletes	K-LCHF	4–6 weeks	HCD	2 (*n* = 15)	SMD	-2.65 (-3.77 to -1.54)	< .001	0.8	Moderate	N/A
Lee et al. 2021 [17]	Total cholesterol, mg/dL	Athletes	K-LCHF	11–24 weeks	RD	2 (*n* = 41)	MD	1.32 (0.64,1.99)	< .001	0	Moderate	No (10.05 mg/dL)

*Abbreviations*: *GRADE* Grading of Recommendations, Assessment, Development, and Evaluations, *HbA*_*1c*_ hemoglobin A_1c_, *HCD* high carbohydrate diet, *K-LCHF* ketogenic low-carbohydrate high-fat diet, *KD* ketogenic diet, *LCD* low-calorie diet, *LDL-C* low-density lipoprotein cholesterol, *LFD* low-fat diet, *MAD* modified Atkins diet, *MD* mean difference, *MCID* minimally clinically important difference, *N/A* not applicable, *RD* regular diet, *RR* risk ratio, *SMD* standardized mean difference, *T2DM* type 2 diabetes mellitus, *VLCKD* very low-calorie ketogenic diet

**Fig. 2:**
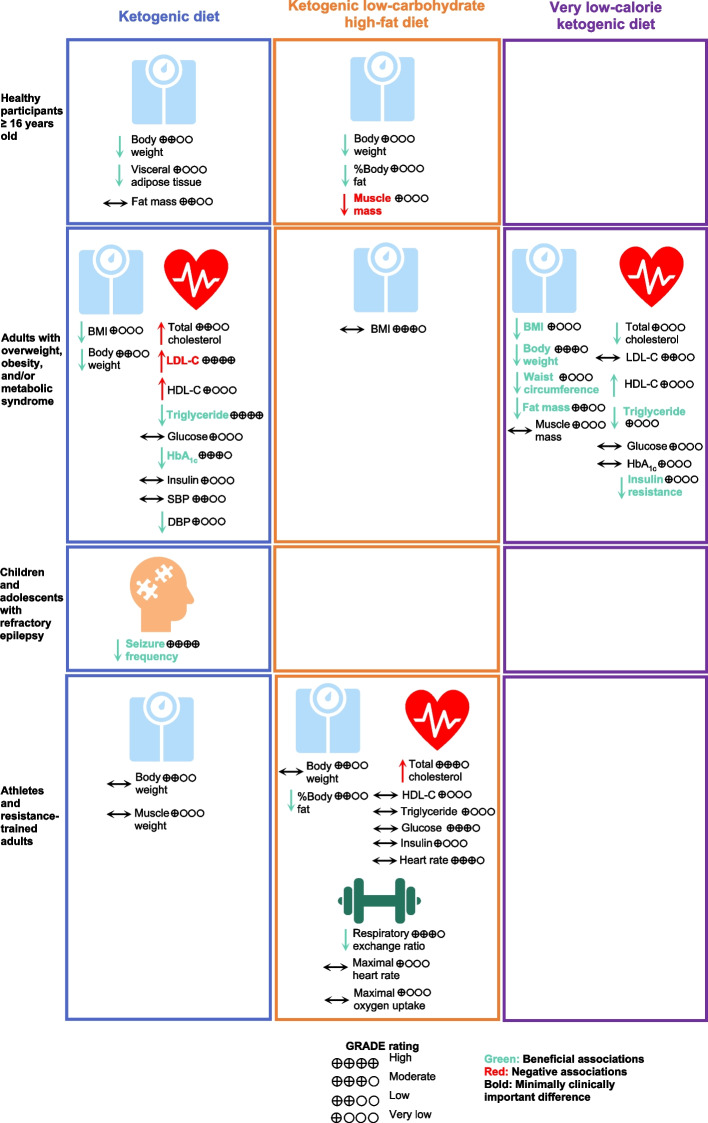
Associations of Types of Ketogenic Diet with Health Outcomes. Abbreviations: BMI, body mass index, DBP, diastolic blood pressure; GRADE, Grading of Recommendations, Assessment, Development, and Evaluations; HbA_1c_, hemoglobin A_1c_; HDL-C, high-density lipoprotein cholesterol; HOMA-IR, homeostatic model of insulin resistance; LDL-C, low-density lipoprotein cholesterol; SBP, systolic blood pressure; TEI, total energy intake

Types of KD showed different effects on health outcomes with changes more than the MCID thresholds in different populations (Fig. 2). KD or MAD for 3–16 months was associated with a 5-times higher proportion of children and adolescents with refractory epilepsy achieving seizure frequency reduction ≥ 50% from baseline compared with a regular diet (RR, 5.11; 95% CI, 3.18 to 8.21) [16]. In healthy participants, K-LCHF for 3–12 weeks could reduce body weight by 3.68 kg (95% CI, -4.45 to -2.90) but also significantly reduced muscle mass by 1.27 kg (95% CI, -1.83 to -0.70, MCID threshold 1.10 kg) [14, 26, 38]. In adults with T2DM, KD for 3–12 months was found to have significant associations with changes more than the MCID thresholds, including reduction of triglyceride and HbA_1c_; however, KD for 12 months led to a clinically meaningful increase in LDL-C by 6.35 mg/dL (95% CI, 2.02 to 10.69, MCID threshold 3.87 mg/dL) [14, 35]. In adults with overweight or obesity and/or metabolic syndrome, VLCKD for 4–6 weeks demonstrated a clinically meaningful weight loss of 9.33 kg (95% CI, -15.45 to -3.22, MCID threshold 4.40 kg) [14, 15]. VLCKD for 3–96 weeks led to a clinically meaningful improvement in BMI, body weight, waist circumference, triglyceride, fat mass, and insulin resistance, while preserving muscle mass [42].

### Sensitivity analyses

Excluding RCTs with small sizes in 7 associations found that the strength of evidence of one association was downgraded to very low quality, i.e., KD for 12 months, and the increase of LDL-C in adults with T2DM compared with a control diet. Another association was downgraded to low quality, i.e., KD for 12 months and the reduction of triglyceride in adults with T2DM compared with the control diet (Additional file 2: Table S6). The remaining associations retained the same rank.

## Discussion

This umbrella review was performed to systematically assess the potential associations of KD and health outcomes by summarizing the evidence from meta-analyses of RCTs. Sensitivity analyses were performed to provide additional evidence from high-quality RCTs, which further increased the reliability of results. We identified 115 associations of KD with a wide range of outcomes. Most associations were rated as low and very low evidence according to the GRADE criteria because of serious imprecision and large heterogeneity in findings, and indirectness due to a mix of different interventions and comparators.

Our findings showed that KD or MAD resulted in better seizure control in children and adolescents with medication-refractory epilepsy (approximately a third of cases) for up to 16 months [10, 11, 16]. Anti-epileptic mechanisms of KD remain unknown but are likely multifactorial. Enhanced mitochondrial metabolism and an increase in ketone bodies or reduction in glucose across the blood–brain barrier resulted in synaptic stabilization [48–50]. Other mechanisms include an increase in gamma-aminobutyric acid (GABA) [51], more beneficial gut microbiome [52], less pro-inflammatory markers [53], and epigenetic modifications (e.g. beta-hydroxybutyrate [beta-OHB]) [54].

In adults, KD was associated with improved anthropometric measures, cardiometabolic parameters, and exercise performance. Our findings, however, demonstrated differences in the level of associations with type of KD. On the one hand, VLCKD is very effective in producing weight loss while preserving muscle mass in adults with overweight or obesity, with specific benefits on anthropometric and cardiometabolic parameters [15, 42]. On the other hand, a significant portion of the weight loss seen in K-LCHF was due to muscle mass loss [17, 38]. Overall KD was negatively associated with reduced muscle mass and increased LDL-C and total cholesterol.

Our findings demonstrated that KD could induce a rapid weight loss in the initial phase of 6 months, after which time further weight loss was hardly achieved [35]. Furthermore, weight loss induced by KD is relatively modest and appears comparable to other dietary interventions that are effective for short-term weight loss, e.g., intermittent fastingand Mediterranean diet [55–57].

KD is one of the dietary interventions employed by individuals to achieve rapid weight loss, which usually comes with reduced muscle mass [58]. However, KD has been hypothesized to preserve muscle mass following weight loss based on several mechanisms, including the protective effect of ketones and its precursors on muscle tissue [59–61], and increased growth hormone secretion stimulated by low blood glucose to increase muscle protein synthesis [58, 62, 63].

With regards to KD effects on lipid profiles, our results demonstrate an effective reduction in serum triglyceride levels with 3 months of lowered dietary carbohydrate intake, with even further reduction by month 12 [35]. Triglyceride levels are consistently shown to decrease after KD. Acute ketosis (beta-OHB ≈ 3 mM) due to ketone supplementation also shows decreases in triglycerides, indicating a potential effect of ketones on triglycerides independent of weight loss. One possible mechanism is the decreased very low-density lipoprotein content in the plasma due to low insulin levels. Due to a lack of insulin, lipolysis increases in fat cells [2, 13, 15]. Of note, the converse has also been observed as a phenomenon known as carbohydrate-induced hypertriglyceridemia, whereby higher dietary carbohydrate intake leads to higher serum triglycerides levels, potentially mediated by changes in triglyceride clearance and hepatic de novo lipogenesis rates [64]. Though our aggregate results also confirm an increase in LDL-C and total cholesterol with KD and K-LCHF, respectively, it is important to note that an increase in either of these levels does not necessarily signify a potentially deleterious cardiovascular end-point. This qualification derives from the fact that LDL particles are widely heterogeneous in composition and size, with small dense LDL particles being significantly more atherogenic than larger LDL particles [65]. Our observed aggregate effect of KD on cholesterol levels does not account for the difference in LDL particle size, nor does it distinguish the sources of dietary fat, which can also be a significant effector of LDL particle size distribution and metabolism [66].

Most RCTs of KD were conducted in patients with a limited group of participants, such as those with overweight, obesity, metabolic syndrome, cancer, and refractory epilepsy. In addition, most outcomes measured were limited to only surrogate outcomes. Thus, more clinical trials with a broader scope in populations and outcomes associated with KD would expand the role of KD in a clinical setting. For example, participant selection could be expanded from previous trials to include elderly patients, nonalcoholic fatty live disease (NAFLD) patients, and polycystic ovarian syndrome patients. Outcomes of interest of could be expanded to include (1) clinical outcomes such as cardiovascular events and liver outcomes, (2) short- and long-term safety outcomes such as adverse events (e.g., gastrointestinal, neurological, hepatic, and renal), eating disorder syndrome, sleep parameters, lipid profiles, and thyroid function and (3) other outcomes such as adherence and quality of life. More importantly, long-term studies are needed to investigate the sustainability of the clinical benefits of KD.

Our findings are useful to support the generation of evidence-based recommendations for clinicians contemplating use of KD in their patients, as well as for the general population. We further emphasize the importance of consultation with healthcare professionals before utilizing KD and any other dietary interventions. We demonstrated the benefits of KD on various outcomes in the short term. However, these improvements may prove difficult to sustain in the long term because of challenges in adherence. As for any diet interventions to achieve sustainable weight loss, factors of success include adherence, negative energy balance, and high-quality foods. Thus, communication and education with KD practitioners are important to ensure their adherence to the diet. Some individuals might benefit from switching from KD to other dietary interventions to maintain long-term weight loss.

### Limitations

This umbrella review has several limitations. Firstly, we focused on published meta-analyses which confined us from assessing the associations of KD on outcomes and populations that were not included in existing meta-analyses. Secondly, most of the included meta-analyses were rated with AMSTAR-2 as critically low confidence, mainly due to a lack of study exclusion reasons, unexplained study heterogeneity, and unassessed publication bias. However, these domains unlikely affected our findings. Thirdly, we could not perform a dose–response analysis to understand the effects of different levels of carbohydrate intake on health outcomes because of insufficient details of carbohydrate intake reported in the meta-analyses. Fourthly, most RCTs of KD were limited to a relatively small number of participants with a short-term follow-up period, which limited our assessment of sustained beneficial effects after stopping KD. Lastly, due to decreased adherence, carbohydrate intake most likely increased across the course of the trials. For example, subjects in the KD arm of the A TO Z Weight Loss Study [67], started with a carbohydrate intake < 10 g/day but ended at 12 months with a carbohydrate intake accounting for 34% of TEI. In the DIRECT trial, subjects in the KD group started with carbohydrate intake of 20 g/day and ended at 12 months with 40% of TEI from carbohydrate intake [68]. Thus, we cannot be certain how the precise degree of ketosis contributed to the beneficial effects noted.

## Conclusions

Beneficial associations of practicing KD were supported by moderate- to high-quality evidence, including weight loss, lower triglyceride levels, decreased HbA_1c_, RER, and decreased seizure frequency. However, KD was associated with a clinically meaningful increase in LDL-C. Clinical trials with long-term follow-up are warranted to investigate whether these short-term effects of KD will translate to beneficial effects on more long-term clinical outcomes such as cardiovascular events and mortality.

## Supplementary Information

**Additional file 1.** PRISMA 2020 Main Checklist. 

**Additional file 2:** **Method S1.** Data extraction. **Table S1.** Difference from original review protocol. **Table S2.** Search strategy. **Table S3.** Excluded studies with reasons. **Table S4.** Quality assessment. **Table S5.** Summary of associations. **Table S6.** Sensitivity analyses. 

## Data Availability

All data generated or analysed during this study are included in this published article and its supplementary information files.

## Abbreviations

Beta-OHB: Beta-hydroxybutyrate
BMI: Body mass index
DBP: Diastolic blood pressure
GABA: Gamma-aminobutyric acid
HDL-C: High-density lipoprotein cholesterol
HbA_1c_: Hemoglobin A_1c_
HOMA-IR: Homeostatic model assessment of insulin resistance
K-LCHF: Ketogenic low-carbohydrate high-fat diet
KD: Ketogenic diets
LDL-C: Low-density lipoprotein cholesterol
MAD: Modified Atkins diet
MCID: Minimally clinically important difference
NAFLD: Nonalcoholic fatty liver disease
RCT: Randomized clinical trials
RER: Respiratory exchange ratio
SBP: Systolic blood pressure
T2DM: Type 2 diabetes mellitus
TEI: Total energy intake
VLCKD: Very low-calorie ketogenic diet
VO_2_ max: Maximal oxygen consumption
