# Supplementary material for: Effects of ketogenic diet on health outcomes: an umbrella review of meta-analyses of randomized clinical trials
Source: BMC Med. 2023 May 25;21:196. doi: 10.1186/s12916-023-02874-y (PMC10210275; doi:10.1186/s12916-023-02874-y)
Supplement: Supplementary file 2 — Additional file 2: Method S1. Data extraction. Table S1. Difference from original review protocol. Table S2. Search strategy. Table S3. Excluded studies with reasons. Table S4. Quality assessment. Table S5. Summary of associations. Table S6. Sensitivity analyses. [file 12916_2023_2874_MOESM2_ESM.docx]

**ADDITIONAL FILE 2**

**Effects of Ketogenic Diet on Health Outcomes: an Umbrella review of Meta-analyses of Randomized Clinical Trials**

**Method S1. Data Extraction**

Two reviewers (C.P. and P.S.) independently performed data extraction using a pilot-tested data extraction sheet that was developed and pilot-tested on five randomly selected eligible articles and then refined until finalization. Discrepancies were resolved with consensus by a discussion with the third reviewer (S.K.V.).

We extracted the following data from the eligible articles: first author’s name, publication year, characteristics of participants, description of KD, description of the comparator(s), duration of follow-up, duration of the diet, number of included studies, number of participants, outcomes, study-specific effect sizes (mean difference (MD), standard mean difference (SMD), risk ratio (RR)) and their corresponding confidence intervals and *P* values.

**Table S1. Difference from original review protocol**

| **Original review protocol** | **Difference** | **Rationale** |
| --- | --- | --- |
| We will search PubMed, EMBASE, Epistemonikos and Cochrane, database of systematic reviews from inception to ***May 20th, 2022***, to identify meta-analyses of Randomized clinical trials. | We searched PubMed, EMBASE, Epistemonikos, and the Cochrane database of systematic reviews (CDSR) from the database inception to ***February 15, 2023***. | Update search to include recent publications |

**Table S2. Search Strategy From Database Inception to February 15, 2023 for Meta-Analyses of Randomized Controlled Trials**

| **Database** | **Search term** | **Results** |
| --- | --- | --- |
| Pubmed | (“Ketogenic” OR “ketogenic diet” OR “Very low carbohydrate diet” OR “Very low-carbohydrate diet” OR “Very low carbohydrate ketogenic diet” OR “Very low-carbohydrate ketogenic diet” OR “VLCKD” OR “High-fat diet” OR “High fat diet” OR “Low-carbohydrate diet” OR “Low carbohydrate diet” OR “Very low-calorie ketogenic diet” OR “Very low calorie ketogenic diet” OR “classic ketogenic diet” OR “Long-chain triglyceride diet” OR “Long chain triglyceride diet” OR “LCT diet” OR “Medium chain triglyceride ketogenic diet” OR “Medium-chain triglyceride ketogenic diet” OR “MCT diet” OR “Modified Atkins diet” OR “Low glycemic index treatment” OR “LGIT”) AND (“systematic review” OR “systematic literature review” OR “meta-analysis” OR “meta-analyses” OR “meta analysis” OR “meta analyses”) | 420 |
| EMBASE | (‘Ketogenic’ OR ‘ketogenic diet’ OR ‘Very low carbohydrate diet’ OR ‘Very low-carbohydrate diet’ OR ‘Very low carbohydrate ketogenic diet’ OR ‘Very low-carbohydrate ketogenic diet’ OR ‘VLCKD’ OR ‘High-fat diet’ OR ‘High fat diet’ OR ‘Low-carbohydrate diet’ OR ‘Low carbohydrate diet’ OR ‘Very low-calorie ketogenic diet’ OR ‘Very low calorie ketogenic diet’ OR ‘classic ketogenic diet’ OR ‘Long-chain triglyceride diet’ OR ‘Long chain triglyceride diet’ OR ‘LCT diet’ OR ‘Medium chain triglyceride ketogenic diet’ OR ‘Medium-chain triglyceride ketogenic diet’ OR ‘MCT diet’ OR ‘Modified Atkins diet’ OR ‘Low glycemic index treatment’ OR ‘LGIT’) AND ('systematic review' OR 'systematic literature review' OR 'meta-analysis' OR 'meta-analyses' OR 'meta analysis' OR 'meta analyses') AND [embase]/lim NOT ([embase]/lim AND [medline]/lim) | 854 |
| Epistemonikos | (title:(Ketogenic AND Meta-analysis) OR abstract:(Ketogenic AND Meta-analysis)) | 67 |
| Cochrane  Database of Systematic Review | (“Ketogenic” OR “ketogenic diet” OR “Very low-carbohydrate diet” OR “VLCKD” OR “High-fat diet” OR “High fat diet” OR “Low-carbohydrate diet” OR “Low carbohydrate diet” OR “Very low-calorie ketogenic diet” OR “Very low calorie ketogenic diet”) | 66 |
|  | **TOTAL** | **1407** |

**Table S3. Excluded Studies With Reasons From the Search for Meta-Analyses of Randomized Controlled Trial**

| **Reasons for exclusion** | **References** |
| --- | --- |
| Not a meta-analysis of ketogenic diet (n= 44) | 1. Ajala O, English P, Pinkney J. Systematic review and meta-analysis of different dietary approaches to the management of type 2 diabetes1-3. American Journal of Clinical Nutrition. 2013;97(3):505-16. 2. Chawla S, Tessarolo Silva F, Amaral Medeiros S, Mekary RA, Radenkovic D. The Effect of Low-Fat and Low-Carbohydrate Diets on Weight Loss and Lipid Levels: A Systematic Review and Meta-Analysis. Nutrients. 2020;12(12). 3. Dong T, Guo M, Zhang P, Sun G, Chen B. The effects of low-carbohydrate diets on cardiovascular risk factors: A meta-analysis. PloS one. 2020;15(1):e0225348. 4. Falkenhain K, Roach LA, McCreary S, McArthur E, Weiss EJ, Francois ME, et al. Effect of carbohydrate-restricted dietary interventions on LDL particle size and number in adults in the context of weight loss or weight maintenance: A systematic review and meta-analysis. American Journal of Clinical Nutrition. 2021;114(4):1455-66. 5. Fechner E, Smeets ETHC, Schrauwen P, Mensink RP. The effects of different degrees of carbohydrate restriction and carbohydrate replacement on cardiometabolic risk markers in humans—a systematic review and meta-analysis. Nutrients. 2020;12(4). 6. Gibson AA, Seimon RV, Lee CMY, Ayre J, Franklin J, Markovic TP, et al. Do ketogenic diets really suppress appetite? A systematic review and meta-analysis. Obesity Reviews. 2015;16(1):64-76. 7. Gjuladin-Hellon T, Davies IG, Penson P, Baghbadorani RA. Effects of carbohydrate-restricted diets on low-density lipoprotein cholesterol levels in overweight and obese adults: A systematic review and meta-analysis. Nutrition reviews. 2019;77(3):161-80. 8. Goldenberg JZ, Day A, Brinkworth GD, Sato J, Yamada S, Jönsson T, et al. Efficacy and safety of low and very low carbohydrate diets for type 2 diabetes remission: systematic review and meta-analysis of published and unpublished randomized trial data. BMJ (Clinical research ed). 2021;372:m4743. 9. Haghighatdoost F, Salehi-Abargouei A, Surkan PJ, Azadbakht L. The effects of low carbohydrate diets on liver function tests in nonalcoholic fatty liver disease: A systematic review and meta-analysis of clinical trials. Journal of research in medical sciences : the official journal of Isfahan University of Medical Sciences. 2016;21:53. 10. Hashimoto Y, Fukuda T, Oyabu C, Tanaka M, Asano M, Yamazaki M, et al. Impact of low-carbohydrate diet on body composition: meta-analysis of randomized controlled studies. Obesity reviews : an official journal of the International Association for the Study of Obesity. 2016;17(6):499-509. 11. Hu J, Wang Z, Lei B, Li J, Wang R. Effects of a Low-Carbohydrate High-Fat Diet Combined with High-Intensity Interval Training on Body Composition and Maximal Oxygen Uptake: A Systematic Review and Meta-Analysis. International journal of environmental research and public health. 2021;18(20). 12. Hu T, Mills KT, Yao L, Demanelis K, Eloustaz M, Yancy WS, et al. Effects of low-carbohydrate diets versus low-fat diets on metabolic risk factors: A meta-analysis of randomized controlled clinical trials. American Journal of Epidemiology. 2012;176(SUPPL. 7):S44-S54. 13. Huntriss R, Campbell M, Bedwell C. The interpretation and effect of a low-carbohydrate diet in the management of type 2 diabetes: a systematic review and meta-analysis of randomised controlled trials. European journal of clinical nutrition. 2018;72(3):311-25. 14. Jayedi A, Zeraattalab-Motlagh S, Jabbarzadeh B, Hosseini Y, Jibril AT, Shahinfar H, et al. Dose-dependent effect of carbohydrate restriction for type 2 diabetes management: a systematic review and dose-response meta-analysis of randomized controlled trials. The American journal of clinical nutrition. 2022. 15. Li S, Ding L, Xiao X. Comparing the Efficacy and Safety of Low-Carbohydrate Diets with Low-Fat Diets for Type 2 Diabetes Mellitus Patients: A Systematic Review and Meta-Analysis of Randomized Clinical Trials. International journal of endocrinology. 2021;2021. 16. Ludwig DS, Dickinson SL, Henschel B, Ebbeling CB, Allison DB. Do Lower-Carbohydrate Diets Increase Total Energy Expenditure? An Updated and Reanalyzed Meta-Analysis of 29 Controlled-Feeding Studies. The Journal of nutrition. 2021;151(3):482-90. 17. Lyons L, Schoeler NE, Langan D, Cross JH. Use of ketogenic diet therapy in infants with epilepsy: A systematic review and meta-analysis. Epilepsia. 2020;61(6):1261-81. 18. Mansoor N, Vinknes KJ, Veierod MB, Retterstol K. Effects of low-carbohydrate diets v. low-fat diets on body weight and cardiovascular risk factors a meta-analysis of randomised controlled trials. British Journal of Nutrition. 2016;115(3):466-79. 19. McArdle PD, Greenfield SM, Rilstone SK, Narendran P, Haque MS, Gill PS. Carbohydrate restriction for glycaemic control in Type 2 diabetes: a systematic review and meta-analysis. Diabetic Medicine. 2019;36(3):335-48. 20. Meng Y, Bai H, Wang S, Li Z, Wang Q, Chen L. Efficacy of low carbohydrate diet for type 2 diabetes mellitus management: A systematic review and meta-analysis of randomized controlled trials. Diabetes research and clinical practice. 2017;131:124-31. 21. Naude CE, Brand A, Schoonees A, Nguyen KA, Chaplin M, Volmink J. Low-carbohydrate versus balanced-carbohydrate diets for reducing weight and cardiovascular risk. Cochrane Database of Systematic Reviews. 2022;2022(1). 22. Naude CE, Schoonees A, Senekal M, Young T, Garner P, Volmink J. Low carbohydrate versus isoenergetic balanced diets for reducing weight and cardiovascular risk: A systematic review and meta-analysis. PloS one. 2014;9(7). 23. Nicholas AP, Soto-Mota A, Lambert H, Collins AL. Restricting carbohydrates and calories in the treatment of type 2 diabetes: a systematic review of the effectiveness of 'low-carbohydrate' interventions with differing energy levels. Journal of nutritional science. 2021;10:e76. 24. Oyabu C, Hashimoto Y, Fukuda T, Tanaka M, Asano M, Yamazaki M, et al. Impact of low-carbohydrate diet on renal function: a meta-analysis of over 1000 individuals from nine randomised controlled trials. The British journal of nutrition. 2016;116(4):632-8. 25. Rezaei S, Abdurahman AA, Saghazadeh A, Badv RS, Mahmoudi M. Short-term and long-term efficacy of classical ketogenic diet and modified Atkins diet in children and adolescents with epilepsy: A systematic review and meta-analysis. Nutritional neuroscience. 2019;22(5):317-34. 26. Sackner-Bernstein J, Kanter D, Kaul S. Dietary intervention for overweight and obese adults: comparison of low- carbohydrate and low-fat diets. a meta- analysis. PloS one. 2015;10(10). 27. Santos FL, Esteves SS, da Costa Pereira A, Yancy WS, Jr., Nunes JP. Systematic review and meta-analysis of clinical trials of the effects of low carbohydrate diets on cardiovascular risk factors. Obesity reviews : an official journal of the International Association for the Study of Obesity. 2012;13(11):1048-66. 28. Schwingshackl L, Chaimani A, Schwedhelm C, Toledo E, Pünsch M, Hoffmann G, et al. Comparative effects of different dietary approaches on blood pressure in hypertensive and pre-hypertensive patients: A systematic review and network meta-analysis. Critical reviews in food science and nutrition. 2019;59(16):2674-87. 29. Suyoto PST. Effect of low-carbohydrate diet on markers of renal function in patients with type 2 diabetes: A meta-analysis. Diabetes/metabolism research and reviews. 2018;34(7):e3032. 30. Tobias DK, Chen M, Manson JE, Ludwig DS, Willett W, Hu FB. Effect of low-fat diet interventions versus other diet interventions on long-term weight change in adults: a systematic review and meta-analysis. The lancet Diabetes & endocrinology. 2015;3(12):968-79. 31. Van Zuuren EJ, Fedorowicz Z, Kuijpers T, Pijl H. Effects of low-carbohydrate- compared with low-fat-diet interventions on metabolic control in people with type 2 diabetes: A systematic review including GRADE assessments. American Journal of Clinical Nutrition. 2018;108(2):300-31. 32. Whittaker J, Harris M. Low-carbohydrate diets and men's cortisol and testosterone: Systematic review and meta-analysis. Nutrition and health. 2022:2601060221083079. 33. Willems AEM, Sura-de Jong M, van Beek AP, Nederhof E, van Dijk G. Effects of macronutrient intake in obesity: a meta-analysis of low-carbohydrate and low-fat diets on markers of the metabolic syndrome. Nutrition reviews. 2021;79(4):429-44. 34. Yang Q, Lang X, Li W, Liang Y. The effects of low-fat, high-carbohydrate diets vs. low-carbohydrate, high-fat diets on weight, blood pressure, serum liquids and blood glucose: a systematic review and meta-analysis. European journal of clinical nutrition. 2022;76(1):16-27. 35. Yuan X, Wang J, Yang S, Gao M, Cao L, Li X, et al. Effect of the ketogenic diet on glycemic control, insulin resistance, and lipid metabolism in patients with T2DM: a systematic review and meta-analysis. Nutrition & diabetes. 2020;10(1):38. 36. Zhang X, Zheng Y, Guo Y, Lai Z. The Effect of Low Carbohydrate Diet on Polycystic Ovary Syndrome: A Meta-Analysis of Randomized Controlled Trials. International journal of endocrinology. 2019;2019:4386401. 37. Zhang Y, Yang Y, Huang Q, Zhang Q, Li M, Wu Y. The effectiveness of lifestyle interventions for diabetes remission on patients with type 2 diabetes mellitus: A systematic review and meta-analysis. Worldviews Evid Based Nurs. 2023;20(1):64-78. 38. Morshedzadeh N, Ahmadi AR, Tahmasebi R, Tavasolian R, Heshmati J, Rahimlou M. Impact of low-carbohydrate diet on serum levels of leptin and adiponectin levels: a systematic review and meta-analysis in adult. J Diabetes Metab Disord. 2022;21(1):979-90. 39. Snetselaar LG, Cheek JJ, Fox SS, Healy HS, Schweizer ML, Bao W, et al. Efficacy of Diet on Fatigue and Quality of Life in Multiple Sclerosis: A Systematic Review and Network Meta-analysis of Randomized Trials. Neurology. 2023;100(4):e357-e66. 40. Boyle BR, Ablett AD, Ochi C, Hudson J, Watson L, Rauh D, et al. The effect of weight loss interventions for obesity on fertility and pregnancy outcomes: A systematic review and meta-analysis. Int J Gynaecol Obstet. 2022. 41. Apekey TA, Maynard MJ, Kittana M, Kunutsor SK. Comparison of the Effectiveness of Low Carbohydrate Versus Low Fat Diets, in Type 2 Diabetes: Systematic Review and Meta-Analysis of Randomized Controlled Trials. Nutrients. 2022;14(20). 42. Zhao H, Jin H, Xian J, Zhang Z, Shi J, Bai X. Effect of Ketogenic Diets on Body Composition and Metabolic Parameters of Cancer Patients: A Systematic Review and Meta-Analysis. Nutrients. 2022;14(19). 43. Choi JH, Cho YJ, Kim HJ, Ko SH, Chon S, Kang JH, et al. Effect of Carbohydrate-Restricted Diets and Intermittent Fasting on Obesity, Type 2 Diabetes Mellitus, and Hypertension Management: Consensus Statement of the Korean Society for the Study of Obesity, Korean Diabetes Association, and Korean Society of Hypertension. J Obes Metab Syndr. 2022;31(2):100-22. 44. Yu Y, Huang J, Chen X, Fu J, Wang X, Pu L, et al. Efficacy and Safety of Diet Therapies in Children With Autism Spectrum Disorder: A Systematic Literature Review and Meta-Analysis. Frontiers in Neurology. 2022;13. |
| Not a meta-analysis of randomized controlled trials (n= 23) | 1. Acharya P, Acharya C, Thongprayoon C, Hansrivijit P, Kanduri SR, Kovvuru K, et al. Incidence and Characteristics of Kidney Stones in Patients on Ketogenic Diet: A Systematic Review and Meta-Analysis. Diseases (Basel, Switzerland). 2021;9(2). 2. Coleman JL, Carrigan CT, Margolis LM. Body composition changes in physically active individuals consuming ketogenic diets: a systematic review. Journal of the International Society of Sports Nutrition. 2021;18(1):41. 3. Crawford P, Paden SL. What is the dietary treatment for low HDL cholesterol? Journal of Family Practice. 2006;55(12):1076-8. 4. Henderson CB, Filloux FM, Alder SC, Lyon JL, Caplin DA. Efficacy of the ketogenic diet as a treatment option for epilepsy: meta-analysis. Journal of child neurology. 2006;21(3):193-8. 5. Jirapinyo P, Devery A, Sarker S, Williams G, Thompson CC. A Comparison of Diet Plan Outcomes in Diabetes Management: A Systematic Review and Meta-Analysis. Gastroenterology. 2018;154(6):S-1057-S-8. 6. Li HF, Zou Y, Ding G. Therapeutic Success of the Ketogenic Diet as a Treatment Option for Epilepsy: a Meta-analysis. Iranian journal of pediatrics. 2013;23(6):613-20. 7. Prezioso G, Carlone G, Zaccara G, Verrotti A. Efficacy of ketogenic diet for infantile spasms: A systematic review. Acta neurologica Scandinavica. 2018;137(1):4-11. 8. Wu YC, Zheng D, Sun JJ, Zou ZK, Ma ZL. Meta-analysis of studies on breast cancer risk and diet in Chinese women. International journal of clinical and experimental medicine. 2015;8(1):73-85. 9. Ye F, Li XJ, Jiang WL, Sun HB, Liu J. Efficacy of and patient compliance with a ketogenic diet in adults with intractable epilepsy: a meta-analysis. Journal of clinical neurology (Seoul, Korea). 2015;11(1):26-31. 10. Koerich ACC, Borszcz FK, Thives Mello A, de Lucas RD, Hansen F. Effects of the ketogenic diet on performance and body composition in athletes and trained adults: a systematic review and Bayesian multivariate multilevel meta-analysis and meta-regression. Crit Rev Food Sci Nutr. 2022:1-26. 11. Li M, Yuan J. Effects of very low-carbohydrate ketogenic diet on lipid metabolism in patients with type II diabetes mellitus: a meta-analysis. Nutr Hosp. 2022;39(4):916-23. 12. Luo W, Zhang J, Xu D, Zhou Y, Qu Z, Yang Q, et al. Low carbohydrate ketogenic diets reduce cardiovascular risk factor levels in obese or overweight patients with T2DM: A meta-analysis of randomized controlled trials. Front Nutr. 2022;9:1092031. 13. Mhanna A, Mhanna M, Beran A, Al-Chalabi M, Aladamat N, Mahfooz N. Modified Atkins diet versus ketogenic diet in children with drug-resistant epilepsy: A meta-analysis of comparative studies. Clin Nutr ESPEN. 2022;51:112-9. 14. Taftian M, Beigrezaei S, Arabi V, Salehi-Abargouei A. The Effect of Ketogenic Diet on Weight Loss in Adult Patients with Cancer: A Systematic Review and Meta-Analysis of Controlled Clinical Trials. Nutrition and cancer. 2022:1-13. 15. Uhomoibhi TO, Okobi TJ, Okobi OE, Koko JO, Uhomoibhi O, Igbinosun OE, et al. High-Fat Diet as a Risk Factor for Breast Cancer: A Meta-Analysis. Cureus. 2022;14(12):e32309. 16. Xiao Y, Xue K, Dang Z, Wang M, He G, Guo H. Efficacy and safety of a very low-calorie ketogenic diet (VLCKD) in patients with overweight and obesity: a meta-analysis. Chinese Journal of Evidence-Based Medicine. 2022;22(4):403-10. 17. Zaki HA, Iftikhar H, Abdalrubb A, Al-Marri NDR, Abdelrahim MG, Fayed M, et al. Clinical Assessment of Intermittent Fasting With Ketogenic Diet in Glycemic Control and Weight Reduction in Patients With Type II Diabetes Mellitus: A Systematic Review and Meta-Analysis. Cureus. 2022;14(10):e30879. 18. Zaki HA, Iftikhar H, Bashir K, Gad H, Samir Fahmy A, Elmoheen A. A Comparative Study Evaluating the Effectiveness Between Ketogenic and Low-Carbohydrate Diets on Glycemic and Weight Control in Patients With Type 2 Diabetes Mellitus: A Systematic Review and Meta-Analysis. Cureus. 2022;14(5):e25528. 19. Amanollahi A, Khazdouz M, Malekahmadi M, Klement RJ, Lee D, Khodabakhshi A. Effect of Ketogenic Diets on Cardio-Metabolic Outcomes in Cancer Patients: A Systematic Review and Meta-Analysis of Controlled Clinical Trials. Nutr Cancer. 2023;75(1):95-111. 20. Furini C, Spaggiari G, Simoni M, Greco C, Santi D. Ketogenic state improves testosterone serum levels-results from a systematic review and meta-analysis. Endocrine. 2023;79(2):273-82. 21. Membrilla JA, Roa J, Díaz-de-Terán J. Preventive treatment of refractory chronic cluster headache: systematic review and meta-analysis. J Neurol. 2023;270(2):689-710. 22. Varaee H, Darand M, Hassanizadeh S, Hosseinzadeh M. Effect of low-carbohydrate diet on depression and anxiety: A systematic review and meta-analysis of controlled trials. J Affect Disord. 2023;325:206-14. 23. Du Y, Oh C, No J. Effects of the ketogenic diet on components of the metabolic syndrome: A systematic review and meta-analysis. Nutrition Clinique et Metabolisme. 2023;37(1):10-20. |
| Not a meta-analysis with the largest dataset (n = 2) | 1. Parry-Strong A, Wright-McNaughton M, Weatherall M, Hall RM, Coppell KJ, Barthow C, et al. Very low carbohydrate (ketogenic) diets in type 2 diabetes: A systematic review and meta-analysis of randomized controlled trials. Diabetes Obes Metab. 2022;24(12):2431-42. 2. Zhou C, Wang M, Liang J, He G, Chen N. Ketogenic Diet Benefits to Weight Loss, Glycemic Control, and Lipid Profiles in Overweight Patients with Type 2 Diabetes Mellitus: A Meta-Analysis of Randomized Controlled Trails. Int J Environ Res Public Health. 2022;19(16). |

**Table S4. Quality Assessment of the Included Studies Using A Measurement Tool to Assess Systematic Reviews (AMSTAR-2)**

| **Author** | **AMSTAR-2** | | | | | | | | | | | | | | | | | |
| --- | --- | --- | --- | --- | --- | --- | --- | --- | --- | --- | --- | --- | --- | --- | --- | --- | --- | --- |
|  | **Item 1** | **Item 2*** | **Item 3** | **Item 4*** | **Item 5** | **Item 6** | **Item 7*** | **Item 8** | **Item 9*** | **Item 10** | **Item 11*** | **Item 12** | **Item 13*** | **Item 14** | **Item 15*** | **Item 16** |  | **Overall** |
| Alarim et al, 2020 |  |  |  |  |  |  |  |  |  |  |  |  |  |  |  |  |  | **CL** |
| Amini et al, 2021 |  |  |  |  |  |  |  |  |  |  |  |  |  |  |  |  |  | **M** |
| Ashtary-Larky et al, 2022 |  |  |  |  |  |  |  |  |  |  |  |  |  |  |  |  |  | **CL** |
| Bueno et al, 2013 |  |  |  |  |  |  |  |  |  |  |  |  |  |  |  |  |  | **M** |
| Cao et al, 2021 |  |  |  |  |  |  |  |  |  |  |  |  |  |  |  |  |  | **CL** |
| Castellana et al, 2020 |  |  |  |  |  |  |  |  |  |  |  |  |  |  |  |  |  | **L** |
| Choi et al, 2020 |  |  |  |  |  |  |  |  |  |  |  |  |  |  |  |  |  | **CL** |
| Lee et al, 2021 |  |  |  |  |  |  |  |  |  |  |  |  |  |  |  |  |  | **CL** |
| Lee et al, 2021 |  |  |  |  |  |  |  |  |  |  |  |  |  |  |  |  |  | **CL** |
| Lopez-Espinosa et al, 2021 |  |  |  |  |  |  |  |  |  |  |  |  |  |  |  |  |  | **CL** |
| Muscogiuri et al, 2021 |  |  |  |  |  |  |  |  |  |  |  |  |  |  |  |  |  | **CL** |
| Rafiullah et al, 2022 |  |  |  |  |  |  |  |  |  |  |  |  |  |  |  |  |  | **CL** |
| Sainsbury et al, 2018 |  |  |  |  |  |  |  |  |  |  |  |  |  |  |  |  |  | **L** |
| Smith et al, 2020 |  |  |  |  |  |  |  |  |  |  |  |  |  |  |  |  |  | **CL** |
| Sourbron et al, 2020 |  |  |  |  |  |  |  |  |  |  |  |  |  |  |  |  |  | **CL** |
| Vargas-Molina et al, 2022 |  |  |  |  |  |  |  |  |  |  |  |  |  |  |  |  |  | **CL** |
| Yang et al, 2021 |  |  |  |  |  |  |  |  |  |  |  |  |  |  |  |  |  | **CL** |

|  |
| --- |
|  |
|  |

Yes H: High L: Low

Partial Yes M: Moderate CL: Critically low

No

**Item 1.** Did the research questions and inclusion criteria for the review include the components of PICO?

**Item 2.** Did the report of the review contain an explicit statement that the review methods were established prior to the conduct of the review
and did the report justify any significant deviations from the protocol?*

**Item 3.** Did the review authors explain their selection of the study designs for inclusion in the review?

**Item 4.** Did the review authors use a comprehensive literature search strategy?*

**Item 5.** Did the review authors perform study selection in duplicate?

**Item 6.** Did the review authors perform data extraction in duplicate?

**Item 7.** Did the review authors provide a list of excluded studies and justify the exclusions?*

**Item 8.** Did the review authors describe the included studies in adequate detail?

**Item 9.** Did the review authors use a satisfactory technique for assessing the risk of bias (RoB) in individual studies that were included in the review?*

**Item 10.** Did the review authors report on the sources of funding for the studies included in the review?

**Item 11.** If meta-analysis was performed did the review authors use appropriate methods for statistical combination of results?*

**Item 12.** If meta-analysis was performed, did the review authors assess the potential impact of RoB in individual studies on the results of the meta-analysis or other evidence synthesis?

**Item 13.** Did the review authors account for RoB in individual studies when interpreting/ discussing the results of the review?*

**Item 14.** Did the review authors provide a satisfactory explanation for, and discussion of, any heterogeneity observed in the results of the review?

**Item 15.** If they performed quantitative synthesis did the review authors carry out an adequate investigation of publication bias (small study bias) and discuss its likely impact on the results of the review?*

**Item 16.** Did the review authors report any potential sources of conflict of interest, including any funding they received for conducting the review?

**Critical domain*

**Table S5. Summary of Associations of Ketogenic Diet with Health Outcomes in Different Populations**

| Source | Outcome | Population | Intervention | Duration of KD | Comparator | No. of studies (sample size) | Metric | Random effect size (95% CI) | *P* value | *I^2^* , % | GRADE rating | Clinical importance of significant association (MCID threshold) |
| --- | --- | --- | --- | --- | --- | --- | --- | --- | --- | --- | --- | --- |
| Healthy participants | | | | | | | | | | | | |
| Amini et al, 2021 | Body weight, kg | Healthy adults | KD | 8-12 weeks | RD | 3 (n=105) | MD | **-2.95  (-5.26 to -0.64)** | **.012** | 1.1 | Low | No (MCID 4.40 kg) |
| Amini et al, 2021 | Fat mass, kg | Healthy participants | KD | 8-12 weeks | LFD, LCD, HCD, or RD | 2 (n=45) | MD | -0.79  (-1.99 to 0.40) | .19 | 0.3 | Low |  |
| Participants ≥ 16 years old | | | | | | | | | | | | |
| Ashtary-Larky et al, 2022 | Body fat, % | Individuals ≥ 16 years old | K-LCHF | 3-10 weeks | RD | 5 (n=100) | MD | **-2.27  (-3.64 to -0.91)** | **.001** | 80.0 | Very low | No (MCID 5%) |
| Ashtary-Larky et al, 2022 | Body weight, kg | Individuals ≥ 16 years old | K-LCHF | 3-12 weeks | RD | 13 (n=244) | MD | **-3.68  (-4.45 to -2.90)** | **<.001** | 18.1 | Very low | No (MCID 4.40 kg) |
| Ashtary-Larky et al, 2022 | Muscle mass, kg | Individuals ≥ 16 years old | K-LCHF | 3-12 weeks | RD | 13 (n=244) | MD | **-1.27  (-1.83 to -0.70)** | **<.001** | 23.0 | Very low | Yes (MCID 1.10 kg) |
| Amini et al, 2021 | Visceral adipose tissue, g | Adults ≥18 years old | KD | 8-12 weeks | RD | 2 (n=206) | MD | **-28.91  (-50.75 to -7.24)** | **.009** | 0 | Very low | N/A |
| Amini et al, 2021 | BMI, kg/m^2^ | Adults ≥ 18 years old | KD or K-LCHF | 2-52 weeks | LFD, LCD, HCD, or RD | 8 (n=723) | MD | **-1.44  (-2.07 to -1.89)** | **<.001** | 75.9 | Very low | No (MCID 2.00 kg/m^2^) |
| Amini et al, 2021 | Body weight, kg | Adults ≥ 18 years old | KD or K-LCHF | 2-96 weeks | LFD, LCD, HCD, or RD | 18 (n=1653) | MD | **-2.87  (-3.84 to -1.89)** | **<.001** | 68.7 | Very low | No (MCID 4.40 kg) |
| Amini et al, 2021 | Fat mass, kg | Adults ≥18 years old | KD or K-LCHF | 2-96 weeks | LFD, LCD, HCD, or RD | 11 (n=937) | MD | **-1.40  (-2.50 to -0.30)** | **.012** | 64.0 | Very low | No (MCID 3.30 kg) |
| Amini et al, 2021 | Muscle mass, kg | Adults ≥ 18 years old | KD or K-LCHF | 4-52 weeks | LFD, LCD, HCD, or RD | 6 (n=386) | MD | **-0.81  (-1.32 to -0.30)** | **.002** | 0 | Very low | No (MCID 1.10 kg) |
| Amini et al, 2021 | Waist circumference, cm | Adults ≥ 18 years old | KD or K-LCHF | 2-24 weeks | LFD, LCD, or HCD | 3 (n=599) | MD | **-3.23  (-4.38 to -2.09)** | **<.001** | 0 | Low | No (MCID 5.0 cm) |
| Adults with overweight, obesity, or metabolic syndrome | | | | | | | | | | | | |
| Bueno et al, 2013 | Body weight, kg | Adults with obesity | KD | 12-24 weeks | LFD or RD | 13 (n=1569) | MD | **-0.91  (-1.65 to -0.17)** | **.02** | 0 | Low | No (MCID 4.40 kg) |
| Bueno et al, 2013 | SBP, mmHg | Adults with obesity | KD | 12-24 weeks | LFD or RD | 11 (n=1298) | MD | -1.47  (-3.44 to 0.50) | .14 | 33.0 | Low |  |
| Bueno et al, 2013 | DBP, mmHg | Adults with obesity | KD | 12-24 weeks | LFD or RD | 11 (n=1298) | MD | **-1.43  (-2.49 to -0.37)** | **.008** | 3.0 | Very low | No (MCID 2.0 mmHg) |
| Bueno et al, 2013 | HDL-C, mg/dL | Adults with obesity | KD | 12-24 weeks | LFD or RD | 12 (n=1258) | MD | **1.62  (1.08 to 2.16)** | **<.001** | 9.0 | Low | No (MCID 3.87 mg/dL) |
| Bueno et al, 2013 | LDL-C, mg/dL | Adults with obesity | KD | 12-24 weeks | LFD or RD | 12 (n=1258) | MD | **2.16  (0.72 to 3.6)** | **.002** | 0 | Very low | No (MCID 3.87 mg/dL) |
| Bueno et al, 2013 | TG, mg/dL | Adults with obesity | KD | 12-24 weeks | LFD or RD | 12 (n=1258) | MD | **-3.24  (-4.86 to -1.44)** | **<.001** | 12.0 | Very low | No (MCID 7.96 mg/dL) |
| Lopez-Esponosa et al, 2021 | BMI, kg/m^2^ | Adults with obesity | K-LCHF | 6-24 months | HCD | 2 (n=230) | MD | -0.06  (-0.21 to 0.34) | .69 | 0.1 | Moderate |  |
| Lopez-Esponosa et al, 2021 | TC, mg/dL | Adults with obesity | KD, K-LCHF, or VLCKD | 6-24 months | LFD, LCD, or HCD | 4 (n=624) | MD | -0.06  (-0.64 to 0.52) | .83 | 59.5 | Very low |  |
| Lopez-Esponosa et al, 2021 | HDL-C, mg/dL | Adults with obesity | KD, K-LCHF, or VLCKD | 6-24 months | LFD, LCD, or HCD | 4 (n=624) | MD | .04  (-0.84 to 0.93) | .92 | 96.2 | Very low |  |
| Lopez-Esponosa et al, 2021 | LDL-C, mg/dL | Adults with obesity | KD, K-LCHF, or VLCKD | 6-24 months | LFD, LCD, or HCD | 4 (n=624) | MD | -0.12  (-0.49 to 0.26) | .55 | 25.6 | Very low |  |
| Lopez-Esponosa et al, 2021 | TG, mg/dL | Adults with obesity | KD, K-LCHF, or VLCKD | 6-24 months | LFD, LCD, or HCD | 4 (n=624) | MD | -0.28  (-0.83 to 0.27) | .32 | 54.1 | Very low |  |
| Lee et al, 2021 | Body weight, kg | Adults with overweight or obesity | KD | 6-10 weeks | RD | 2 (n=53) | MD | -0.77  (-2.45 to 0.90) | .90 | 83.0 | Very low |  |
| Lee et al, 2021 | VO_2_ max, ml/kg/min | Adults with overweight or obesity | KD | 1-6 months | RD | 3 (n=139) | MD | .18  (-0.17 to 0.41) | .43 | 0 | Low |  |
| Amini et al, 2021 | Body weight, kg | Adults with overweight or obesity | KD | 2-24 weeks | LFD | 2 (n=239) | MD | -4.03  (-7.57 to 0.49) | .26 | 54.9 | Very low |  |
| Choi et al, 2020 | TC, mg/dL | Adults with overweight or obesity | KD | 1-12 months | LFD, LCD, or RD | 4 (n=235) | MD | **0.34  (0.08 to 0.61)** | **.01** | 11.0 | Low | No (10.05 mg/dL) |
| Choi et al, 2020 | HDL-C, mg/dL | Adults with overweight or obesity | KD | 1-12 months | LFD, LCD, or RD | 4 (n=235) | MD | 0.08  (-0.18 to 0.34) | .53 | 52.0 | Very low |  |
| Choi et al, 2020 | LDL-C, mg/dL | Adults with overweight or obesity | KD | 1-12 months | LFD, LCD, or RD | 4 (n=235) | MD | **0.35  (0.09 to 0.61)** | **.009** | 0 | Low | No (MCID 3.87 mg/dL) |
| Choi et al, 2020 | TG, mg/dL | Adults with overweight or obesity | KD | 1-12 months | LFD, LCD, or RD | 4 (n=235) | MD | -0.05  (-0.31 to 0.21) | .70 | 0 | Low |  |
| Amini et al, 2021 | BMI, kg/m^2^ | Adults with overweight or obesity | KD | 2-24 weeks | LFD, LCD, HCD, or RD | 5 (n=148) | MD | -0.87  (-2.08 to 0.34) | .16 | 11.9 | Very low |  |
| Choi et al, 2020 | FPG, mg/dL | Adults with overweight or obesity | KD | 1-12 months | LFD, LCD, HCD, or RD | 6 (n=290) | MD | -0.35  (-0.76 to 0.07) | .07 | 54.0 | Very low |  |
| Choi et al, 2020 | Insulin, mIU/dL | Adults with overweight or obesity | KD | 1-12 months | LFD, LCD, HCD, or RD | 3 (n=200) | MD | -0.10  (-0.38 to 0.18) | .50 | 80.0 | Very low |  |
| Muscogiuri et al, 2021 | BMI, kg/m^2^ | Adults with overweight or obesity | VLCKD | 3-96 weeks | LCD | 4 (n=216) | MD | **-2.78  (-4.62 to -0.93)** | **.003** | 38.6 | Very low | Yes (MCID 2.00 kg/m^2^) |
| Muscogiuri et al, 2021 | Body weight, kg | Adults with overweight or obesity | VLCKD | 3-96 weeks | LCD | 5 (n=227) | MD | **-8.18  (-13.07 to -3.29)** | **.001** | 96.2 | Very low | Yes (MCID 4.40 kg) |
| Muscogiuri et al, 2021 | Muscle mass, kg | Adults with overweight or obesity | VLCKD | 3-96 weeks | LCD | 3 (n=127) | MD | -0.23  (-5.19 to 0.03) | .93 | 16.7 | Very low |  |
| Muscogiuri et al, 2021 | Waist circumference, cm | Adults with overweight or obesity | VLCKD | 3-96 weeks | LCD | 4 (n=216) | MD | **-8.33  (-11.34 to -5.33)** | **<.001** | 92.0 | Very low | Yes (MCID 5.0 cm) |
| Muscogiuri et al, 2021 | TC, mg/dL | Adults with overweight or obesity | VLCKD | 3-96 weeks | LCD | 3 (n=198) | MD | **-7.13  (-9.71 to -4.55)** | **<.001** | 51.0 | Very low | No (10.05 mg/dL) |
| Muscogiuri et al, 2021 | HDL-C, mg/dL | Adults with overweight or obesity | VLCKD | 3-96 weeks | LCD | 3 (n=198) | MD | **3.14  (0.70 to 5.59)** | **.01** | 84.0 | Very low | No (MCID 3.87 mg/dL) |
| Muscogiuri et al, 2021 | LDL-C, mg/dL | Adults with overweight or obesity | VLCKD | 3-96 weeks | LCD | 3 (n=127) | MD | -5.96  (-13.42 to 1.50) | .12 | 90.0 | Very low |  |
| Muscogiuri et al, 2021 | TG, mg/dL | Adults with overweight or obesity | VLCKD | 3-96 weeks | LCD | 3 (n=198) | MD | **-29.9  (-42.47 to -17.32)** | **<.001** | 89.0 | Very low | Yes (MCID 7.96 mg/dL) |
| Muscogiuri et al, 2021 | Fat mass, kg | Adults with overweight or obesity | VLCKD | 3-96 weeks | LCD | 3 (n=127) | MD | **-12.20  (-13.90 to -10.50)** | **<.001** | 6.6 | Low | Yes (MCID 3.30 kg) |
| Muscogiuri et al, 2021 | FPG, mg/dL | Adults with overweight or obesity | VLCKD | 3-96 weeks | LCD | 3 (n=198) | MD | -4.43  (-11.39 to 2.52) | .21 | 94.0 | Very low |  |
| Muscogiuri et al, 2021 | HbA_1c_, % | Adults with overweight or obesity | VLCKD | 3-96 weeks | LCD | 3 (n=198) | MD | -0.23  (-0.54 to 0.07) | .14 | 97.0 | Very low |  |
| Muscogiuri et al, 2021 | HOMA-IR | Adults with overweight or obesity | VLCKD | 3-96 weeks | LCD | 2 (n=145) | MD | **-1.49  (-2.86 to -0.13)** | **.032** | 93.0 | Very low | Yes (MCID 0.05) |
| Amini et al, 2021 | Body weight, kg | Adults with overweight or obesity | KD or K-LCHF | 2-96 weeks | LFD, LCD, HCD, or RD | 8 (n=535) | MD | -1.61  (-3.85 to 0.62) | .62 | 32.2 | Very low |  |
| Amini et al, 2021 | Fat mass, kg | Adults with overweight or obesity | KD or K-LCHF | 2-96 weeks | LFD, LCD, HCD, or RD | 7 (n=311) | MD | -0.86  (-2.42 to 0.71) | .28 | 18.0 | Very low |  |
| Amini et al, 2021 | Muscle mass, kg | Adults with overweight or obesity | KD or K-LCHF | 4-24 weeks | LFD, LCD, HCD, or RD | 4 (n=127) | MD | -0.50  (-1.50 to 0.49) | .32 | 1.2 | Very low |  |
| Smith et al, 2020 | Body weight, kg | Adults with obesity, some with dyslipidemia | KD | 3-24 months | LFD, RD | 5 (n=447) | MD | -1.69  (-4.76 to 1.38) | .64 | 79.9 | Very low |  |
| Choi et al, 2020 | TG, mg/dL | Adults with overweight or obesity, some with T2DM | KD or K-LCHF | 4 days to 24 months | LFD, LCD, or HCD | 7 (n=403) | MD | **-0.45  (-0.80 to -0.10)** | **.01** | 59.0 | Very low | No (MCID 7.96 mg/dL) |
| Choi et al, 2020 | DBP, mmHg | Adults with overweight or obesity, some with T2DM | KD or K-LCHF | 3-12 months | LFD, LCD, HCD, or RD | 6 (n=451) | MD | -0.60  (-0.49 to 0.16) | .31 | 58.0 | Very low |  |
| Choi et al, 2020 | SBP, mmHg | Adults with overweight or obesity, some with T2DM | KD or K-LCHF | 3-12 months | LFD, LCD, HCD, or RD | 6 (n=451) | MD | -0.10  (-0.43 to 0.24) | .57 | 61.0 | Very low |  |
| Choi et al, 2020 | C-reactive protein, mg/dL | Adults with overweight or obesity, some with T2DM | KD or K-LCHF | 3-12 months | LFD or HCD | 4 (n=329) | MD | 0.02  (-0.20 to 0.23) | .87 | 0 | Low |  |
| Choi et al, 2020 | Serum creatinine , mg/dL | Adults with overweight or obesity, some with T2DM | KD or K-LCHF | 6-24 months | LFD, LCD, or HCD | 4 (n=338) | MD | 0.11  (-0.11 to 0.32) | .32 | 37.0 | Low |  |
| Choi et al, 2020 | BMI, kg/m^2^ | Adults with overweight or obesity, some with T2DM | KD or VLCKD | 1-12 months | LFD, LCD, RD | 6 (n=297) | MD | -0.63  (-1.35 to 0.08) | .08 | 87.0 | Very low |  |
| Choi et al, 2020 | Body weight, kg | Adults with overweight or obesity, some with T2DM | KD or VLCKD | 1-8 months | LFD, LCD, or RD | 8 (n=485) | MD | -0.46  (-0.90 to 0.03) | **.**05 | 78.0 | Low |  |
| Choi et al, 2020 | Waist circumference, cm | Adults with overweight or obesity, some with T2DM | KD or VLCKD | 1-12 months | LFD or LCD | 5 (n=392) | MD | -0.48  (-1.04 to 0.07) | .09 | 82.0 | Very low |  |
| Choi et al, 2020 | HbA_1c_, % | Adults with overweight or obesity, some with T2DM | KD or VLCKD | 3-12 months | LFD, LCD, or RD | 6 (n=412) | MD | **-0.48  (-0.68 to -0.27)** | **<.001** | 23.0 | Very low | No (MCID 0.50%) |
| Choi et al, 2020 | TC, mg/dL | Adults with overweight or obesity, some with T2DM | KD, K-LCHF, or VLCKD | 1-12 months | LFD, LCD, HCD, or RD | 14 (n=523) | MD | 0.17  (-0.01 to 0.35) | .07 | 14.0 | Low |  |
| Choi et al, 2020 | HDL-C, mg/dL | Adults with overweight or obesity, some with T2DM | KD, K-LCHF, or VLCKD | 1-24 months | LFD, LCD, HCD, or RD | 10 (n=618) | MD | **0.22  (0.05 to 0.38)** | **.01** | 10.0 | Low | No (MCID 3.87 mg/dL) |
| Choi et al, 2020 | LDL-C, mg/dL | Adults with overweight or obesity, some with T2DM | KD, K-LCHF, or VLCKD | 1-24 months | LFD, LCD, HCD, or RD | 10 (n=616) | MD | **0.18  (0.01 to 0.34)** | **.04** | 0 | Low | No (MCID 3.87 mg/dL) |
| Choi et al, 2020 | TG, mg/dL | Adults with overweight or obesity, some with T2DM | KD, K-LCHF, or VLCKD | 4 days to 24 months | LFD, LCD, HCD, or RD | 11 (n=638) | MD | -0.23  (-0.51 to 0.05) | .11 | 60.0 | Very low |  |
| Choi et al, 2020 | FPG, mg/dL | Adults with overweight or obesity, some with T2DM | KD, K-LCHF, or VLCKD | 120 mins to 24 months | LFD, LCD, HCD, or RD | 12 (n=628) | MD | -0.25  (-0.50 to 0.00) | .05 | 52.0 | Very low |  |
| Choi et al, 2020 | HOMA-IR | Adults with overweight or obesity, some with T2DM | KD, K-LCHF, or VLCKD | 120 mins to 12 months | LFD, LCD, HCD, or RD | 5 (n=310) | MD | -0.19  (-0.42 to 0.05) | .12 | 49.0 | Low |  |
| Choi et al, 2020 | Insulin, mIU/dL | Adults with overweight or obesity, some with T2DM | KD, K-LCHF, or VLCKD | 120 mins to 12 months | LFD, LCD, HCD, or RD | 9 (n=531) | MD | -0.04  (-0.22 to 0.13) | .62 | 46.0 | Low |  |
| Rafiullah et al, 2022 | Body weight, kg | Adults with T2DM | KD | 3 months | RD | 6 (n=388) | MD | **-2.91  (-4.88 to -0.95)** | **.004** | 62.0 | Low | No (MCID 4.4 kg) |
| Rafiullah et al, 2022 | Body weight, kg | Adults with T2DM | KD | 6 months | RD | 6 (n=415) | MD | **-2.84  (-5.29 to -0.39)** | **.02** | 79.0 | Very low | No (MCID 4.4 kg) |
| Rafiullah et al, 2022 | Body weight, kg | Adults with T2DM | KD | 12 months | RD | 4 (n=297) | MD | -0.03  (-2.35 to 2.29) | .98 | 59.0 | Very low |  |
| Rafiullah et al, 2022 | LDL-C, mg/dL | Adults with T2DM | KD | 3 months | RD | 3 (n=255) | MD | 0.62  (-2.39 to 3.62) | .69 | 0 | Low |  |
| Rafiullah et al, 2022 | LDL-C, mg/dL | Adults with T2DM | KD | 6 months | RD | 6 (n=415) | MD | 6.34  (-0.87 to 13.56) | .08 | 78.0 | Very low |  |
| Rafiullah et al, 2022 | LDL-C, mg/dL | Adults with T2DM | KD | 12 months | RD | 4 (n=389) | MD | **6.35  (2.02 to 10.69)** | **.004** | 0 | High | Yes (MCID 3.87 mg/dL) |
| Rafiullah et al, 2022 | TG, mg/dL | Adults with T2DM | KD | 3 months | RD | 4 (n=283) | MD | **-18.36  (-24.24 to -12.49)** | **<.001** | 0 | High | Yes (MCID 7.96 mg/dL) |
| Rafiullah et al, 2022 | TG, mg/dL | Adults with T2DM | KD | 6 months | RD | 7 (n=542) | MD | **-20.60  (-41.15 to -0.05)** | **.049** | 84.0 | Very low | Yes (MCID 7.96 mg/dL) |
| Rafiullah et al, 2022 | TG, mg/dL | Adults with T2DM | KD | 12 months | RD | 5 (n=445) | MD | **-24.10  (-33.93 to -14.27)** | **<.001** | 0 | High | Yes (MCID 7.96 mg/dL) |
| Rafiullah et al, 2022 | HbA_1c_, % | Adults with T2DM | KD | 3 months | RD | 6 (n=388) | MD | **-0.61  (-0.82 to -0.40)** | **<.001** | 44.0 | Moderate | Yes (MCID 0.50%) |
| Rafiullah et al, 2022 | HbA_1c_, % | Adults with T2DM | KD | 6 months | RD | 7 (n=542) | MD | **-0.58  (-0.85 to -0.32)** | **<.001** | 56.0 | Low | Yes (MCID 0.50%) |
| Rafiullah et al, 2022 | HbA_1c_, % | Adults with T2DM | KD | 12 months | RD | 5 (n=445) | MD | -0.17  (-0.47 to 0.12) | .26 | 48.0 | Moderate |  |
| Amini et al, 2021 | BMI, kg/m^2^ | T2DM adults with overweight or obesity | KD | 3-6 months | LCD | 2 (n=397) | MD | **-1.94  (-3.02 to -0.87)** | **<.001** | 20.4 | Very low | No (MCID 2.00 kg/m^2^) |
| Alarim et al, 2020 | BMI, kg/m^2^ | T2DM adults with overweight or obesity | KD | 4-6 months | LCD | 2 (n=173) | MD | -1.55  (-4.16 to 1.05) | .24 | 35.9 | Very low |  |
| Alarim et al, 2020 | TG, mg/dL | T2DM adults with overweight or obesity | KD | 4-8 months | LCD | 3 (n=198) | MD | **-1.06  (-1.35 to -0.77)** | **<.001** | 95.0 | Very low | No (MCID 7.96 mg/dL) |
| Sainsbury et al, 2018 | HbA_1c_, % | T2DM Adults with overweight or obesity | KD | 3 months | LCD | 2 (n=118) | MD | **-0.63  (-1.00 to -0.26)** | **<.001** | 0.2 | Low | Yes (MCID 0.50%) |
| Alarim et al, 2020 | Body weight, kg | T2DM adults with overweight or obesity | KD | 4-8 months | LCD or RD | 3 (n=198) | MD | **-4.26  (-6.88 to -1.63)** | **.002** | 81.0 | Very low | No (MCID 4.40 kg) |
| Sainsbury et al, 2018 | HbA_1c_, % | T2DM Adults with overweight or obesity | KD | 6 months | LCD or LFD | 2 (n=216) | MD | **-0.71  (-1.26 to -0.16)** | **.012** | 0.4 | Low | Yes (MCID 0.50%) |
| Choi et al, 2020 | C-peptide, ng/mL | T2DM adults with overweight or obesity | K-LCHF | 120 mins to 4 days | LFD or HCD | 2 (n=74) | MD | -0.20  (-0.66 to 0.25) | .38 | 0 | Low |  |
| Castellana et al, 2020 | Body weight, kg | T2DM adults with overweight or obesity | VLCKD | 4-6 weeks | LFD or RD | 2 (n=142) | MD | **-9.33  (-15.45 to -3.22)** | **<.001** | 0.1 | Moderate | Yes (MCID 4.4 kg) |
| Choi et al, 2020 | TC, mg/dL | T2DM adults with overweight or obesity | KD or K-LCHF | 4-24 months | LFD, LCD, or HCD | 3 (n=288) | MD | 0.01  (-0.24 to 0.26) | .93 | 0 | Low |  |
| Choi et al, 2020 | HDL-C, mg/dL | T2DM Adults with overweight or obesity | KD or K-LCHF | 3-24 months | LFD, LCD, or HCD | 6 (n=381) | MD | **0.31  (0.10 to 0.52)** | **.005** | 0 | Low | No (MCID 3.87 mg/dL) |
| Choi et al, 2020 | LDL-C, mg/dL | T2DM adults with overweight or obesity | KD or K-LCHF | 3-12 months | LFD, LCD, or HCD | 6 (n=301) | MD | 0.06  (-0.15 to 0.28) | .91 | 0 | Low |  |
| Choi et al, 2020 | HOMA-IR | T2DM Adults with overweight or obesity | KD or K-LCHF | 3-24 months | LFD, LCD, or HCD | 4 (n=272) | MD | **-0.29  (-0.54 to -0.04)** | **.02** | 0 | Low | Yes (MCID 0.05) |
| Choi et al, 2020 | HbA_1c_, % | T2DM adults with overweight or obesity | KD or VLCKD | 3-12 months | LFD, LCD, or RD | 5 (n=266) | MD | **-0.62  (-0.89 to -0.35)** | **<.001** | 0 | Low | Yes (MCID 0.50%) |
| Choi et al, 2020 | FPG, mg/dL | T2DM adults with overweight or obesity | KD, K-LCHF, or VLCKD | 120 mins to 24 months | LFD, LCD, HCD, or RD | 6 (n=396) | MD | -0.19  (-0.53 to 0.15) | .28 | 56.0 | Very low |  |
| Choi et al, 2020 | Insulin, mIU/dL | T2DM adults with overweight or obesity | KD, K-LCHF, or VLCKD | 120 mins to 24 months | LFD, LCD, HCD, or RD | 6 (n=341) | MD | -0.01  (-0.24 to 0.22) | .22 | 8.0 | Low |  |
| Children and adolescents with refractory epilepsy | | | | | | | | | | | | |
| Sourbron et al, 2020 | Seizure frequency reduction ≥50% from baseline | Children or adolescents (age 1–18 years) with refractory epilepsy | KD or MAD | 3-16 months | RD | 5 (n=374) | RR | **5.11  (3.18 to 8.21)** | **<.001** | 0 | High | N/A |
| Adults with cancer | | | | | | | | | | | | |
| Yang et al, 2021 | Body weight, kg | Adults with cancers | KD or K-LCHF | 4-24 weeks | RD | 4 (n=151) | MD | -0.34  (-1.33 to 0.65) | .50 | 87.0 | Very low |  |
| Yang et al, 2021 | TC, mg/dL | Adults with cancers | KD or K-LCHF | 4-24 weeks | RD | 3  (n=91) | MD | .25  (-0.17 to 0.67) | .24 | 0 | Low |  |
| Yang et al, 2021 | HDL-C, mg/dL | Adults with cancers | KD or K-LCHF | 4-24 weeks | RD | 3  (n=91) | MD | -0.07  (-0.50 to 0.35) | .74 | 69.0 | Very low |  |
| Yang et al, 2021 | LDL-C, mg/dL | Adults with cancers | KD or K-LCHF | 4-24 weeks | RD | 3  (n=91) | MD | .21  (-0.21 to 0.63) | .32 | 0 | Low |  |
| Yang et al, 2021 | TG, mg/dL | Adults with cancers | KD or K-LCHF | 4-24 weeks | RD | 3  (n=91) | MD | .09  (-0.33 to 0.51) | .68 | 53.0 | Very low |  |
| Yang et al, 2021 | FPG, mg/dL | Adults with cancers | KD or K-LCHF | 4-24 weeks | RD | 4 (n=150) | MD | -0.40  (-1.23 to 0.42) | .34 | 82.0 | Very low |  |
| Yang et al, 2021 | Insulin, mIU/dL | Adults with cancers | KD or K-LCHF | 4-24 weeks | RD | 3  (n=90) | MD | 0.11  (-1.33 to 1.55) | .88 | 89.0 | Very low |  |
| Yang et al, 2021 | Adverse events | Adults with cancers | KD or K-LCHF | 4-24 weeks | RD | 2 (n=329) | RR | 1.27  (0.29 to 5.47) | .75 | 82.0 | Very low |  |
| Athletes or resistance-trained adults | | | | | | | | | | | | |
| Lee et al, 2021 | Body weight, kg | Athletes | K-LCHF | 2-11 weeks | RD | 4  (n=76) | MD | -0.09  (-0.55 to 0.36) | .29 | 59.0 | Low |  |
| Lee et al, 2021 | Body fat, % | Athletes | K-LCHF | 2-24 weeks | RD | 3  (n=51) | MD | **-0.69  (-1.26 to -0.13)** | **.016** | 0 | Low | No (MCID 5%) |
| Lee et al, 2021 | TC, mg/dL | Athletes | K-LCHF | 11-24 weeks | RD | 2  (n=41) | MD | **1.32  (0.64,1.99)** | **<.001** | 0 | Moderate | No (10.05 mg/dL) |
| Lee et al, 2021 | HDL-C, mg/dL | Athletes | K-LCHF | 1-6 months | RD | 3  (n=57) | MD | 1.07  (-0.21 to 2.35) | .10 | 79.0 | Very low |  |
| Lee et al, 2021 | TG, mg/dL | Athletes | K-LCHF | 1-6 months | RD | 3  (n=57) | MD | -0.49  (-2.58 to 1.61) | .65 | 95.0 | Very low |  |
| Lee et al, 2021 | FPG, mg/dL | Athletes | K-LCHF | 11-24 weeks | RD | 2  (n=41) | MD | -0.13  (-0.82 to 0.58) | .70 | 0 | Moderate |  |
| Lee et al, 2021 | Insulin, mIU/dL | Athletes | K-LCHF | 11-24 weeks | RD | 2  (n=41) | MD | -0.19  (-1.14 to 0.76) | .69 | 70.0 | Very low |  |
| Lee et al, 2021 | Heart rate, bpm | Athletes | K-LCHF | 1-6 months | RD | 2  (n=32) | MD | 0.02  (-0.68 to 0.72) | .95 | 0 | Moderate |  |
| Cao et al, 2021 | Maximal heart rate, bpm | Athletes | K-LCHF | 4-6 weeks | HCD | 2  (n=15) | MD | 0.21  (-0.49 to 0.90) | .56 | 0.3 | Very low |  |
| Cao et al, 2021 | Respiratory exchange ratio | Athletes | K-LCHF | 4-6 weeks | HCD | 2  (n=15) | SMD | **-2.65  (-3.77 to -1.54)** | **<.001** | 0.8 | Moderate | N/A |
| Lee et al, 2021 | Respiratory exchange ratio | Athletes | K-LCHF | 1-6 months | RD | 2  (n=32) | SMD | **-1.26  (-2.15 to -0.38)** | **.005** | 94.0 | Very low | N/A |
| Cao et al, 2021 | VO_2_ max, ml/kg/min | Athletes | K-LCHF | 4-6 weeks | HCD | 2  (n=15) | MD | 0.00  (-0.67 to 0.67) | .99 | 0 | Very low |  |
| Lee et al, 2021 | VO_2_ max, ml/kg/min | Athletes | K-LCHF | 4-6 weeks | HCD | 2  (n=32) | MD | -0.05  (-0.75 to 0.64) | .88 | 0 | Moderate |  |
| Vargas-Molina et al, 2022 | Body weight, kg | Athletes or resistance trained adults | KD | 8-12 weeks | RD | 5 (n=96) | MD | 0.31  (-3.15 to 3.77) | 0.86 | 0 | Low |  |
| Vargas-Molina et al, 2022 | Muscle mass, kg | Athletes or resistance trained adults | KD | 8-12 weeks | RD | 4 (n=71) | MD | -0.57  (-3.35 to 2.20) | 0.69 | 0 | Very low |  |

Abbreviations: BMI, body mass index; DBP, diastolic blood pressure; FPG, fasting plasma glucose; GRADE, Grading of Recommendations, Assessment, Development, and Evaluations; HbA_1c_, hemoglobin A_1c_; HCD, high carbohydrate diet; HDL-C, high-density lipoprotein cholesterol; HOMA-IR, homeostatic model of insulin resistance; K-LCHF, ketogenic low-carbohydrate high-fat diet; KD, ketogenic diet; LCD, low-calorie diet; LDL-C, low-density lipoprotein cholesterol; LFD, low-fat diet; MD, mean difference; RD, regular diet; RR, risk ratio; MAD, modified Atkins diet; N/A, not applicable; SMD, standardized mean difference; T2DM, type 2 diabetes mellitus; TC, total cholesterol; TG, triglyceride; VLCKD, very low-calorie ketogenic diet; VO_2_ max, maximal oxygen uptake.

**Table S6. Summary of Sensitivity Analyses of Associations with Moderate- to High-Quality of Evidence**

| **Outcomes** | **Population** | **Duration of KD** | **Intervention** | **Comparator** | **Metric** | **Primary analysis** | | | **Sensitivity analysis: Excluding studies with high risk of bias** | | | **Sensitivity analysis: Excluding studies with small sample size (25^th^ percentile)** | | |
| --- | --- | --- | --- | --- | --- | --- | --- | --- | --- | --- | --- | --- | --- | --- |
|  |  |  |  |  |  | **No. of studies** | **Random effect size (95% CI)** | **GRADE** | **No. of studies** | **Random effect size (95% CI)** | **GRADE** | **No. of studies** | **Random effect size (95% CI)** | **GRADE** |
| **Anthropometric measures** | | | | | | | | | | | | | | |
| Body weight, kg in Castellana et al, 2020 | T2DM adults with overweight or obesity | 4-6 weeks | VLCKD | LFD or RD | MD | 2 | -9.33  (-15.45, -3.22) | Moderate | N/A – remaining studies are not enough to conduct meta-analysis | | | N/A – remaining studies are not enough to conduct meta-analysis | | |
| BMI, kg/m^2^ in Lopez-Espinosa et al, 2021 | Adults with obesity | 6-24 months | K-LCHF | HCD | MD | 2 | 0.06  (-0.21, 0.16) | Moderate | N/A – remaining studies are not enough to conduct meta-analysis | | | 2 | 0.06  (-0.21, 0.16) | Moderate |
| **Lipid profile** | | | | | | | | | | | | | | |
| LDL-C, mg/dl in Rafiullah et al, 2022 | Adults with T2DM | 12 months | KD | RD | MD | 4 | 6.35  (2.02, 10.69) | High | N/A – remaining studies are not enough to conduct meta-analysis | | | 3 | 3.07  (-3.62, 9.75) | Very low |
| Total cholesterol, mg/dL in Lee et al, 2021 | Athletes | 11-24 weeks | K-LCHF | RD | MD | 2 | 1.32  (0.64, 1.99) | Moderate | N/A – remaining studies are not enough to conduct meta-analysis | | | N/A – remaining studies are not enough to conduct meta-analysis | | |
| Triglyceride, mg/dl Rafiullah et al, 2022 | Adults with T2DM | 3 months | KD | RD | MD | 4 | -18.36  (-24.24, -12.49) | High | N/A – remaining studies are not enough to conduct meta-analysis | | | 3 | -18.53  (-26.21, -10.86) | High |
| Triglyceride, mg/dl Rafiullah et al, 2022 | Adults with T2DM | 12 months | KD | RD | MD | 5 | -24.10  (-33.93, -14.27) | High | N/A – remaining studies are not enough to conduct meta-analysis | | | 3 | -19.33  (-41.08, -2.42) | Low |
| **Glycemic profile** | | | | | | | | | | | | | | |
| Fasting plasma glucose , mg/dL in Lee et al, 2021 | Athletes | 11-24 weeks | K-LCHF | RD | MD | 2 | -0.13  (-0.82, 0.58) | Moderate | N/A – remaining studies are not enough to conduct meta-analysis | | | N/A – remaining studies are not enough to conduct meta-analysis | | |
| HbA_1C_, % in Raffiulah et al, 2022 | Adults with T2DM | 3 months | KD | RD | MD | 6 | -0.61  (-0.82, -0.40) | Moderate | N/A – remaining studies are not enough to conduct meta-analysis | | | 4 | -0.59  (-0.86 to -0.31) | Moderate |
| HbA_1C_, % in Raffiulah et al, 2022 | Adults with T2DM | 12 months | KD | RD | MD | 5 | -0.17  (-0.47, 0.12) | Moderate | N/A – remaining studies are not enough to conduct meta-analysis | | | 4 | -0.03  (-0.30 to 0.23) | Moderate |
| **Blood pressure** | | | | | | | | | | | | | | |
| Heart rate, bpm in Lee et al, 2021 | Athletes | 1-6 months | K-LCHF | RD | MD | 2 | 0.02  (-0.68, 0.72) | Moderate | N/A – remaining studies are not enough to conduct meta-analysis | | | N/A – remaining studies are not enough to conduct meta-analysis | | |
| **Seizure** | | | | | | | | | | | | | | |
| Seizure frequency reduction ≥50% from baseline in Sourbron et al, 2020 | Children or adolescents (age 1–18 years) with refractory epilepsy | 3-6 months | KD or MAD | RD | RR | 5 | 5.11  (3.18, 8.21) | High | N/A – remaining studies are not enough to conduct meta-analysis | | | 4 | 3.77  (1.08 to 6.46) | High |
| **Exercise performance** | | | | | | | | | | | | | | |
| Respiratory exchange ratio in Cao et al, 2021 | Athletes | 4-6 weeks | K-LCHF | RD | MD | 2 | -2.66  (-3.77, -1.54) | Moderate | N/A – remaining studies are not enough to conduct meta-analysis | | | N/A – remaining studies are not enough to conduct meta-analysis | | |
| VO2 max, ml/kg/min in Lee et al, 2021 | Athletes | 1-6 months | KD | RD | MD | 2 | -0.05 (-0.75, 0.64) | Moderate | N/A – remaining studies are not enough to conduct meta-analysis | | | N/A – remaining studies are not enough to conduct meta-analysis | | |

Abbreviations: BMI, body mass index; GRADE, Grading of Recommendations, Assessment, Development, and Evaluations; HbA_1c_, hemoglobin A_1c_; HCD, high carbohydrate diet; K-LCHF, ketogenic diet with low-carbohydrate and high-fat; KD, ketogenic diet; LDL-C, low-density lipoprotein cholesterol; LFD, low-fat diet; MAD, modified Atkins diet; MD, mean difference; RD, regular diet; RR, risk ratio; T2DM, type 2 diabetic mellitus; VLCKD, very low-calorie ketogenic diet.
